# Living arrangements and chronic disease accumulation among native-born and immigrant older adults in Europe

**DOI:** 10.1016/j.socscimed.2025.117954

**Published:** 2025-03-12

**Authors:** Su Yeon Jang, Anna Oksuzyan, Frank J. van Lenthe, Mikko Myrskylä, Silvia Loi

**Affiliations:** a Max Planck Institute for Demographic Research, Rostock, Germany; b Oxford Institute of Population Ageing, University of Oxford, Oxford, UK; c Department of Public Health, Erasmus MC University Medical Center Rotterdam, Rotterdam, Netherlands; d School of Public Health, Bielefeld University, Bielefeld, Germany; e Helsinki Institute for Demography and Population Health, University of Helsinki, Helsinki, Finland; f Max Planck - University of Helsinki Center for Social Inequalities in Population Health, Rostock, Germany

**Keywords:** Immigrants, Chronic diseases, Living arrangements, Family, Aging, Europe

## Abstract

**Background:**

Who we live with in later life significantly influences the daily care support we receive, potentially moderating chronic disease trajectories. For immigrants, this relationship is further complicated by cultural preferences for certain living arrangements. This study examines the differential role of living arrangements in chronic disease accumulation among native-born and immigrant older adults in Europe.

**Methods:**

Using data from the Survey of Health, Ageing and Retirement in Europe (2006–2022), we analyze how living arrangements moderate the relationship between age, migration background, and the number of chronic diseases. We also conduct stratified analyses based on immigrants’ countries of origin. All models employ inverse probability weights for panel attrition and panel-robust standard errors for longitudinal data. Analyses are conducted separately for men and women.

**Results:**

Immigrants generally have a higher chronic disease burden than native-born individuals, although the size of this disparity varies by living arrangements. Among native-born men and women, living with a partner/spouse or family is related to fewer chronic diseases than living alone. Conversely, among immigrants, these benefits of shared living arrangements are absent for men and weaker for women. We also find that the role of living arrangements in chronic disease accumulation among immigrants varies by their country of origin, as supported by, for instance, a considerable chronic disease burden among immigrant women from low HDI countries who live as a family.

**Conclusion:**

Older immigrants, particularly men from highly developed countries and women from less developed countries, may experience fewer health benefits from living with a partner or with family. Our findings indicate that these immigrant households may have excessive caregiving burdens, which could contribute to unhealthy aging among immigrants in later life.

## 1. Introduction

Many high-income countries worldwide are facing the rapid aging of their populations, leading to growing caregiving needs for older adults (United Nations, 2017b). This unprecedented demand can strain even the most robust welfare systems, including those in several European countries, resulting in the burden of care falling to informal caregivers due to limitations in formal support (Dukhovnov et al., 2022; Suanet et al., 2012). Chronically ill older adults, in particular, frequently rely on their informal care networks – mainly co-residing household members – for medical support and assistance in activities of daily living (Adelman et al., 2014; Schulz et al., 2020; Wolff and Spillman, 2014). Hence, in aging societies, it is of great importance to understand how people’s living arrangements at older ages can translate into long-term health outcomes.

As the number of immigrants has grown, their health has become a significant public health concern across European societies (International Organization for Migration (IOM), 2021; Kristiansen et al., 2016). Scholars have recently investigated how the health of immigrants evolves as they age in a foreign country, especially in European settings, where they often have an initial health advantage but a less favorable trajectory with age compared to the native-born population (Bousmah et al., 2019; Jang et al., 2023; Loi et al., 2024). While less is known about the mechanisms driving these aging disparities, one potential contributor is the different living arrangements of native-born and immigrant individuals. Immigrants face unique circumstances, such as having left many family members in their country of origin and being influenced by culturally preferred forms of living arrangements from their home country (Albertini and Mantovani, 2022; Barbiano et al., 2018; de Valk and Schans, 2008). These circumstances may shape the extent to which living arrangements moderate the long-term health trajectories of immigrants compared to those of their native-born counterparts.

In this study, we explore the differential role of living arrangements in chronic disease accumulation among native-born and immigrant older adults in Europe using population-representative data from the Survey of Health, Ageing and Retirement in Europe (SHARE). Our paper extends the current knowledge in mainly two ways. First, findings from this study provide evidence of whether and how living arrangements moderate the relationship between age and the accumulation of chronic diseases among native-born and immigrant older adults. Second, our origin-specific analyses contribute to a better understanding of whether the role of living arrangements varies across cultures with different expectations for familial arrangements in later life. These insights can help to identify population subgroups with particularly high caregiving burdens.

### 1.1. Living arrangements and chronic disease accumulation

Living arrangements, which refer to with whom individuals live and how they are related to these co-residents, are important determinants for physical and mental health at older ages. Evidence suggests that compared to living alone, living with a partner or family members is generally associated with lower mortality (Lund et al., 2002; Staehelin et al., 2012), better self-rated and mental health (Aranda, 2015; Courtin and Avendano, 2016; Zunzunegui et al., 2001), and reduced chronic diseases (Wang et al., 2022). Hence, along with global population aging, understanding the implications of living arrangements for health trajectories in later life is becoming increasingly important (United Nations, 2017b).

Studies suggest that within the broader context of social relationships, interpersonal connections can either mitigate or exacerbate the detrimental effects of aging on health outcomes (Benson et al., 2019). This moderating role of social relationships in age-related health decline is also observed within shared living arrangements. Particularly for older adults with chronic diseases, co-residing family members can closely monitor the symptoms of their health conditions and promote healthy behaviors, such as having a balanced diet and regular physical exercise and adhering to the prescribed treatment (Gallant et al., 2010; Wolff and Spillman, 2014). At the same time, more intense interactions among family members may negatively affect older adults’ health, exacerbating their health problems and the progression of chronic diseases (Dekhtyar et al., 2019; Olaya et al., 2017; Woods et al., 2020).

The mechanisms through which relationships within shared living arrangements influence healthy aging processes can help to explain disparities in the speed of chronic disease accumulation across different populations. Prior work has shown that living in materially deprived areas, having lower educational attainment, and experiencing occupational stress are related to faster chronic disease accumulation (Dekhtyar et al., 2019; Lyons et al., 2023). These socioeconomically disadvantaged groups are more likely to transition to less favorable living arrangements (e.g., living alone or being institutionalized) at older ages, which suggests that their accelerated accrual of long-term health conditions may stem from the absence or reduction of benefits from supportive living arrangements and robust social relationships with the people with whom they are living.

### 1.2. Living arrangements and health among immigrants

An essential aspect of family solidarity is that the family network is expected to be available to provide needed care and support (Alburez-Gutierrez et al., 2023). Thus, in populations with limited family networks, individuals are less likely to receive support from their family members due to their physical unavailability. Immigrants often face considerable challenges in relocating with their family members (e.g., spouses, children, parents, and other relatives), resulting in a restricted family network in their host country (Barbiano et al., 2018). This limitation in family networks can lead to immigrants having fewer options for shared living arrangements, which are essential sources of informal care at older ages. With fewer family members available for them to live with, the caregiving responsibilities for older immigrants may fall on a smaller number of people, potentially leading to an excessive burden of care in immigrant families (Adelman et al., 2014; Szinovacz and Davey, 2007). This concentration of the caregiving burden can result in reduced quality of care and, consequently, adverse health outcomes for care recipients (Kuzuya et al., 2011).

Importantly, research has shown that immigrants form families in patterns that differ not only from those of native-born individuals but also between men and women (Barbiano et al., 2018; Castro Torres and Gutierrez-Vazquez, 2022). For instance, compared to immigrant men, immigrant women are more likely to arrive in the host country for family reunification purposes and thus have a higher probability of living with family at older ages (Barbiano et al., 2018). Consequently, the excessive caregiving burden within immigrant families may tend to fall on immigrant women, potentially leading to gender disparities in the association between living arrangements and health outcomes among immigrants.

Cultural variations in the common and preferred types of living arrangements can further complicate their implications for immigrants’ health at older ages. In wealthier and more developed countries, older adults usually live in one-generation households rather than in larger families (Reher and Requena, 2018). Multigenerational households and close-knit family living arrangements are more prevalent in less economically developed countries, such as those in Asia, Africa, and parts of Latin America (Esteve and Reher, 2024; Gallant et al., 2010; Reher and Requena, 2018). These differences in prevailing norms across cultures suggest that immigrants from different countries of origin can have widely varying preferences and expectations regarding their living arrangements in later life. It has, for instance, been shown that Turkish and Moroccan immigrants in the Netherlands have stronger expectations of living with their children at older ages than other immigrant groups (de Valk and Schans, 2008). When immigrants’ ideal living arrangements in later life do not match their reality, they may experience diminished well-being and decreased life satisfaction, compromising their physical and mental health at older ages (Aranda, 2015; Tosi and Grundy, 2018).

Several previous studies have observed an initial health advantage for immigrants upon their arrival in the receiving country (Akresh and Frank, 2008; Constant et al., 2017; Ichou and Wallace, 2019; Loi and Hale, 2019; Riosmena et al., 2017). However, this initial advantage tends to diminish over time as immigrants age in the host society (Antecol and Bedard, 2006; Bousmah et al., 2019; Loi et al., 2024; Loi and Hale, 2019; Reiss et al., 2015). This trend of eroding health benefits suggests that immigrants experience rapid health deterioration – or unhealthy aging. This assumption is supported by a recent study showing that in Europe, immigrants accumulate chronic diseases faster with age than native-born individuals (Jang et al., 2023).

One potential mechanism driving inequalities in healthy aging between immigrants and native-born individuals is their differential experiences with various living arrangements. Existing studies on general social relationships have found that immigrants often receive less emotional support from their social networks than native-born individuals, leading to adverse physical and mental health outcomes (Salinero-Fort et al., 2011; Vega et al., 1991; Xu et al., 2017). Although the number of quantitative studies is limited, existing research suggests that living with others may protect older immigrants against depression (Mui, 1999; Wilmoth and Chen, 2003). However, to the best of our knowledge, no previous work has examined whether living arrangements moderate long-term health trajectories, as measured by chronic disease accumulation, more strongly among immigrants than among native-born individuals.

### 1.3. Research aims

In this paper, we aim to comprehensively examine the role of living arrangements – living alone, with a partner, or with family – in shaping the long-term health trajectories of native-born and immigrant older adults in Europe. Our study is guided by the following research question: *Do specific living arrangements play different roles in the age-related accumulation of chronic diseases between native-born and immigrant older adults?* Drawing on literature showing that immigrant households typically have more limited family networks in the host society – a factor that may lead to excessive caregiving burdens and limit the family’s capacity to effectively manage health conditions – we expect to find a smaller protective effect of shared living arrangements against chronic disease accumulation among immigrants than among their native-born counterparts (Barbiano et al., 2018). We also expect the impact of living arrangements on chronic disease accumulation to vary across immigrant groups based on their country of origin. Specifically, we hypothesize that for immigrants from less developed countries (as measured by the Human Development Index), who are more likely to have strong traditions of familial living arrangements, living with a partner or family members is more strongly associated with a slower accumulation of chronic diseases compared to those from more developed countries.

## 2. Methods

### 2.1. Data and study population

We use data from the Survey of Health, Ageing and Retirement in Europe (SHARE), a population-representative longitudinal study of the health and socioeconomic characteristics of older adults in 28 European countries and Israel. Our analysis includes participants from waves 2, 4, 5, 6, 7, 8, 9 (2006–2022), during years in which medical histories for all chronic diseases of interest to us are provided (n = 151,687). We focus on individuals with information on familial relationships, which includes respondents of the cover screen questionnaire in waves 2 through 7, and all respondents in waves 8 and 9 (n = 132,894). We exclude individuals living with non-family members (n = 132,227) due to the high heterogeneity within these non-family groups, which may include friends, ex-partners, or other non-family members (e.g., formal caregivers). Moreover, these individuals comprised only 0.5 % of participants, making detailed analyses infeasible. In order to address the lack of statistical power due to scarce data on immigrants at extreme ages, the sample is limited to participants aged 50–79 (n = 116,809). Furthermore, we maintain our focus on immigrants in Europe by excluding participants from Israel (n = 114,314). After removing respondents with missing information on the outcome and covariates, our final analytic sample includes 112,358 individuals (263,822 observations).

### 2.2. Number of chronic diseases

Our study uses the number of chronic diseases as the outcome measure. We construct this variable using self-reported data on doctor-diagnosed conditions and drug use (detailed information in Table S1). As in prior work, we select 12 chronic diseases that appear with high or medium frequencies in studies on the co-occurrence of multiple chronic diseases: heart attack, stroke, hypertension, diabetes, chronic respiratory diseases, arthritis, osteoporosis, mental disorders, stomach ulcers, Parkinson’s disease, dementia, and cancer (Hafezparast et al., 2021). We combine heart attack and stroke, given that SHARE collects drug usage information that is not differentiated for these two diseases. Since most chronic conditions are considered irreversible, we define individuals who have ever had a condition as prevalent (Griffith et al., 2018). Finally, we count the number of prevalent chronic diseases in each person at each survey wave.

### 2.3. Key independent variables

This study focuses on three key variables: age, migration background, and living arrangements. Age is estimated as a continuous variable using the year and month of birth. We center age at 50 years, the lower bound of the age range in our study. This approach allows the intercept to represent the expected outcome at age 50 rather than zero, which is a more meaningful reference given our focus on older ages. Migration background is determined based on the country of birth, with individuals born in the interview country being classified as native-born and those born elsewhere being classified as immigrants regardless of their current nationality. Living arrangements are grouped into three categories: living alone, living as a couple, and living as a family. Living as a couple is defined as co-residing only with a spouse or partner. Living as a family is defined as co-residing with other family members, such as children, parents, and other relatives, irrespective of the presence of a spouse or partner in the household.

### 2.4. Confounders

Our models include education, income, employment, and parental status to control for potential variations in the outcomes due to differences in socioeconomic and demographic characteristics. Education is categorized into three levels: low (lower secondary education or less), medium (upper secondary or post-secondary non-tertiary education), and high (tertiary education). Household income is divided into low, medium, and high levels using the imputed household net income provided in SHARE, based on tertile cutoffs calculated for each country at each wave. Employment status is categorized into working and not working. Parental status is grouped into two categories: having a child and being childless.

### 2.5. Statistical analysis

Our study applies the weighted least square (WLS) linear regression models, clustering standard errors at the individual level to account for the longitudinal nature of our data. We choose linear regression despite our outcome measure being count data, as it represents a cumulative health state rather than independent events with a defined exposure period, making Poisson regression less appropriate.

As the main focus of our study is how age-related changes in health differ between native-born and immigrant individuals, it is important to deal with different probabilities of panel attrition between the two groups. For instance, immigrants may migrate back to their home countries when they suffer health problems at older ages, resulting in immigrant panels that seemingly remain healthier as they age than their native-born counterparts (Palloni and Arias, 2004). However, it should be noted that return migration alone may not be sufficient in explaining the immigrant health advantage (Abraído-Lanza et al., 1999; Turra and Elo, 2008). To account for the potential differential attrition without imposing restrictive parametric assumptions about the selection mechanism, we apply the inverse probability of censoring weighting approach (Robins et al., 1995). We predict the probability of panel attrition with linear and quadratic terms of age, migration background, and the interaction between migration background and age terms. Final weights are calculated as the cumulative product of the inverse probability of panel participation from the baseline up to the observation time point within each individual.

To comprehensively address our research question, we construct three progressive models (model specifications in Table 1). On the basis of recent findings indicating that the speed of chronic disease accumulation differs between immigrants and native-born individuals, we include migration background, age (linear and quadratic terms), and the interaction between migration background and age in all our models (Jang et al., 2023). We begin by examining the general relationship between living arrangements and chronic disease accumulation at older ages by fitting a model that includes living arrangements in addition to baseline variables (Model 1). Building on this foundation, we assess how living arrangements moderate chronic disease accumulation trajectories, focusing on two aspects. First, we examine whether living arrangements moderate the relationship between being an immigrant and the number of chronic diseases in general – i.e., the intercept of the disease accumulation trajectory – by introducing an interaction term between migration background and living arrangements to the model (Model 2). Second, we test the moderating effects of living arrangements on the speed of chronic disease accumulation – i.e., the slope of the trajectory – specific to an individual’s migration status by adding a three-way interaction between migration background, age terms, and living arrangements to the model (Model 3). Additionally, we include country fixed effects and the survey wave variable to all models to control for unobserved characteristics specific to the country and to the period, respectively. We present all our results in graphical form as the predicted number of chronic diseases to facilitate interpretation, with detailed results provided in the online supplementary material.

Living arrangements at older ages vary considerably between men and women, as the higher mortality among men leads to more women experiencing spousal loss and living alone at older ages (Kamiya, 2024). Therefore, we perform all our analyses for men and women separately. Furthermore, as living arrangements among immigrants can correlate with cultural norms and familial values from their home countries, we explore how the results vary by country of origin. We rerun our final model, Model 3, in stratified samples that include native-born individuals and immigrants from each origin group. Drawing from previous international comparative reports on household formations, we categorize the origin countries according to their level of development, as determined by the Human Development Index (HDI) (Esteve and Reher, 2024; Reher and Requena, 2018). The HDI is commonly used in investigations of country-level determinants of living arrangements, as it collectively measures the wealth, education, and life expectancy of a country, all of which are important correlates of its cultural dynamics. We group countries of origin based on their HDI scores in 2015 into low (HDI <0.7), medium (HDI ≥0.7), and high (HDI ≥0.8) development countries (list of countries in Table S2) (Bousmah et al., 2019; Reher and Requena, 2018).

### 2.6. Sensitivity analysis

We test the sensitivity of our findings in several ways. First, we test the presence of the contemporaneous correlation or reverse causality between living arrangements and the number of chronic diseases by lagging the living arrangements variable by one wave. Second, we perform additional analysis excluding immigrants who have stayed in the receiving country for less than ten years, based on the literature suggesting acculturation to the host society over time as a potential mechanism for health deterioration among immigrants (Reiss et al., 2015). We do not include length of stay in our main models because approximately 97 % of our observations are of immigrants who had been in the receiving country for ten or more years, by when immigrants’ health outcomes had likely converged to those of the native-born population (Loi and Hale, 2019). Third, we use an alternate measure for country of origin groupings based on criticisms of the HDI for giving too little consideration to disparities in national income (Sagar and Najam, 1998). Therefore, we run a sensitivity analysis using the gross national income (GNI) per capita instead of the HDI to group the origin countries. We use the GNI per capita in 2015 to group countries of origin into low- (≤US$ 4035), medium- (>US$ 4035 and ≤ US$ 12,475), and high-income (US$ 12,475) economies, with the cutoff lines being drawn from the World Bank classification system (list of countries in Table S3; detailed composition of origin country groups based on HDI and GNI in Table S4) (United Nations, 2017a). Lastly, we conduct additional analyses where we disaggregate individuals living as a family into those living with versus without a spouse/partner, considering that available social support, care resources, and relationship dynamics may differ between the two groups.

## 3. Results

### 3.1. Descriptive characteristics

Table 2 shows the baseline characteristics of the study population. Compared to the native-born sample (45,643 men and 56,911 women), the immigrant men (4,318) and women (5,544) in the analyses are, on average, younger. Additionally, compared to their native-born counterparts, both the male and the female immigrant populations include higher shares of individuals with high education, low household income, and one or more children and those who are living as a family.

Fig. 1 illustrates the age-specific mean of the number of chronic diseases for all observations included in the analysis. Overall, the average number of chronic diseases increases with age for both men and women, regardless of their migration background and living arrangements. Specifically, compared to their native-born counterparts, immigrant men have a higher number of chronic diseases on average across almost all ages when they are living as a couple. Conversely, native-born and immigrant men who are living alone have a more similar number of chronic diseases. Among women, we observe an immigrant disadvantage at all ages regardless of living arrangements.

### 3.2. Predicted number of chronic diseases

Fig. 2 presents the predicted number of chronic diseases by migration background and living arrangements based on each specified model (model estimates in Table S5; predictions in Table S6). For analyses among men, all models show that native-born men living alone have a higher predicted number of chronic diseases than those living as a couple or a family, while the predicted outcome does not differ significantly among immigrant men regardless of their living arrangements. Model 1 reveals that living as a couple or as a family is related to having fewer chronic diseases than living alone for both native-born and immigrant men, although the benefit is not statistically significant for immigrants. In Model 2, which includes the interaction between migration background and living arrangements, the predicted number of chronic diseases becomes largely similar across living arrangements for immigrant men (alone: 1.84 [1.70–1.98]; couple: 1.88 [1.81–1.95]; family:1.82 [1.71–1.92]), while the benefits of living as a couple or as a family remain for native-born men (alone: 1.79 [1.75–1.83]; couple: 1.68 [1.66–1.70]; family: 1.70 [1.67–1.74]). Finally, we observe only minimal changes in the predicted estimates after adjusting for the three-way interaction between migration background, age, and living arrangements (Model 3).

Analyses for women show that the role of living arrangements in chronic disease accumulation at older ages does not differ significantly between native-born and immigrant women. In all models, the predicted numbers of chronic diseases are lowest for women living as a couple and are highest for women living alone, irrespective of their immigrant history/background. The final model shows that for both immigrant and native-born women, living as a couple (native-born: 1.97 [1.95–1.99]; immigrant: 2.29 [2.22–2.37]) is related to fewer chronic diseases than living alone (native-born: 2.14 [2.11–2.17]; immigrant: 2.47 [2.37–2.27]). Similar to men, we observe a benefit of shared living arrangements for women living with family only among native-born individuals (native-born: 2.05 [2.02–2.08]; immigrant: 2.38 [2.26–2.50]).

### 3.3. Chronic disease accumulation

Fig. 3 extends the findings presented in Fig. 2 by illustrating the age-related trajectories of chronic disease accumulation for men and women by their migration background and living arrangements. Consistent with Figs. 2 and 3 shows that compared to their native-born counterparts, immigrant men living alone have a similar number of chronic diseases regardless of age, while those living as a couple or as a family have a higher number of chronic diseases at almost all ages. Notably, the slopes of disease accumulation trajectories are only marginally different between native-born and immigrant men across all living arrangements. These patterns reveal that living arrangements can moderate the relationship between migration background and the overall number of chronic diseases at older ages, but not the speed at which these conditions are accumulated.

The age-specific predictions for women are also consistent with the findings in Fig. 2, as the predicted number of chronic diseases among immigrants is higher than that among their native-born counterparts regardless of living arrangements at almost all ages. Similar to the findings for men, the chronic disease trajectories of native-born and immigrant women appear to be nearly the same across all living arrangements. As there are only minimal differences in both the general levels and the slopes of the chronic disease accumulation trajectories, our findings suggest that the role of living arrangements in later-life health does not differ significantly between native-born and immigrant women.

### 3.4. Country of origin variations in the chronic disease burden

Fig. 4 presents the variations in the predicted number of chronic diseases by the development level of the origin country, as measured by the HDI (model estimates in Table S7; prediction results in Table S8). Among men, we find no statistically significant differences in the predicted number of chronic diseases across living arrangements for immigrants from both low HDI countries (alone: 2.18 [95 % CI: 1.61–2.74]; couple: 1.67 [1.43–1.92]; family:1.56 [1.23–1.90]) and high HDI countries (alone: 1.78 [1.62–1.94]; couple: 1.91 [1.83–2.00]; family: 1.92 [1.76–2.07]). Among women immigrants, those from low HDI countries are predicted to have a similarly high number of chronic diseases when they are living alone (2.48 [2.09–2.86]) or living in a family (2.54 [2.25–2.83]), while those from high HDI countries are predicted to have the highest number of chronic diseases when they are living alone (2.49 [2.37–2.61]). Additionally, living as a couple is related to fewer chronic diseases, regardless of the country of origin (low HDI countries: 2.06 [1.79–2.32]; medium HDI countries:2.33 [2.16–2.49]; high HDI countries:2.32 [2.23–2.41]).

To further examine how these patterns develop over the years along with age, we examine the age-related trajectories of chronic disease accumulation across different origin countries and living arrangements (Figs. S1–2). Compared to their native-born counterparts, male immigrants across all origin countries accumulate chronic diseases at similar speeds regardless of their living arrangements (Fig. S1). We do, however, observe some differences in the rate of chronic disease accumulation between immigrant women from low and medium HDI countries compared to native-born women (Fig. S2). Specifically, compared to their native-born counterparts, immigrant women from low HDI countries are predicted to accumulate chronic diseases at a faster rate when living with family, while those from medium HDI countries exhibit an accelerated speed of disease accumulation when living as a couple. Conversely, we do not observe such disparities across living arrangements for immigrant women from high HDI countries, as they have a higher number of chronic diseases than native-born women across all living arrangements.

### 3.5. Findings from sensitivity analysis

Our sensitivity analyses support the robustness of our main findings. Analyses using lagged living arrangements reveal chronic disease trajectories consistent with our main findings (Table S9, Fig. S3), as do the analyses excluding immigrants who stayed in the receiving country for less than ten years (Table S10, Fig. S4). Results remain robust when using GNI per capita instead of the HDI to group countries of origin (Table S11, Fig. S5). When distinguishing individuals living as a family with versus without a spouse/partner, the health benefits of the family living arrangements remain evident when they include a spouse/partner (Table S12, Fig. S6). Interestingly, both native-born and immigrant individuals show similar chronic disease burdens when living only with other family members without a spouse/partner and when living alone. Given the very small number of immigrants in these groups, particularly among men, these results should, however, be taken with caution.

## 4. Discussion

### 4.1. Summary of findings

In this study, we examine the role of living arrangements in chronic disease accumulation among native-born and immigrant individuals in Europe. Overall, immigrants have a higher chronic disease burden than native-born individuals. However, this disparity varies depending on living arrangements, with men and women showing different patterns. Our results indicate that for both native-born men and native-born women, living with a partner or in a family is associated with having fewer chronic diseases at older ages, although the advantage is less apparent for women living in a family. For immigrants, however, this protective role of shared living arrangements differs between men and women, remaining robust among women but weakening considerably among men. Consequently, compared to their native-born counterparts, immigrant men living as a couple or as a family are predicted to have more chronic diseases, while immigrant women have a higher disease burden regardless of their living arrangements. The differences in the role of living arrangements in chronic disease accumulation among native-born and immigrant individuals are primarily in the baseline level of chronic diseases, rather than in the speed at which these conditions are accumulated. Our findings further suggest that the role of living arrangements in chronic disease accumulation varies based on the development level of the origin country. Specifically, we show that women from low HDI countries who live as a family have a particularly high chronic disease burden.

### 4.2. Interpretations

As hypothesized, we found that native-born individuals living with a partner or as a family have a lower number of chronic diseases, while the protective effect of having shared living arrangements is lost for immigrants, especially for men. This observation aligns with our theoretical expectation that family support within the household may not provide the same level of health benefits for immigrants as for their native-born counterparts. Our findings are striking, particularly considering that family solidarity and partnership are critical sources of the emotional, instrumental, and financial support immigrants need to navigate the challenges of life in a new country (Creese et al., 2008; Held, 2018; Singh et al., 2015). Indeed, previous studies have observed more frequent intergenerational transfers of care and support among families within immigrant communities than among families of the majority population across Europe (Albertini et al., 2019). We extend the literature by suggesting that living with a partner or family may provide fewer health benefits for immigrants than for their native-born counterparts. Our findings may reflect systemic inequalities faced by immigrants, such as restricted access to health and social care services (Solé-Auró et al., 2012), creating health vulnerabilities that cannot be fully offset by family support systems in later life.

One thing to note is that immigrant women are predicted to have a higher number of chronic diseases than native-born women not only when they are living as a couple or as a family, but also when they are living alone, leading to a consistent immigrant disadvantage for women irrespective of their living arrangements. This persistent disadvantage suggests that living arrangements may play a less significant role in chronic disease accumulation among immigrant women due to the unique challenges they face that transcend the benefits and challenges associated with certain types of living arrangements. Our findings are in line with previous studies showing that immigrant women are less positively selected in terms of health. Research on migration intentions and health outcomes has shown that immigrants with more voluntary motivations for migration, such as pursuing economic or educational opportunities, tend to be more positively selected in terms of health (Moullan and Jusot, 2014). As immigrant women often migrate for family reunification purposes, they may have weaker health selection than men, who are more likely to move for economic and educational purposes (Gkiouleka and Huijts, 2020).

Our study further reveals that the implications of living arrangements for chronic disease accumulation at older ages vary among immigrants depending on their countries of origin. We hypothesized that for immigrants from less developed countries with strong traditions of familial care, there is a stronger protective association between living with a family and chronic disease accumulation than for immigrants from highly developed countries. However, we do not observe statistically significant differences in the chronic disease burden across living arrangements for immigrant men from either low or high HDI countries, although there is a suggestive trend toward lower chronic disease burden among those from low HDI countries when living as couple or family. Moreover, for women, we find a pattern that further diverges from the hypothesis, as, contrary to expectations, immigrant women from low HDI countries are observed to face as high a chronic disease burden when living with family as when living alone. Our findings indicate that the relationship between living arrangements and chronic health outcomes among immigrants varies across countries of origin, with these variations showing different patterns between men and women. This heterogeneity might be due to several factors that we are not able to measure in this study, including varying migration pathways that can significantly impact immigrants’ health profiles (Giuntella et al., 2018), as well as gender- and origin-specific expectations about family roles that can influence both living arrangements and health outcomes (Llácer et al., 2007). Patterns revealed in the current study merit further investigation in future studies to understand their underlying mechanisms.

Our study highlights the need to invest in social support systems that effectively integrate immigrants, rather than assuming that they will solely rely on their informal networks. Immigrant families are less likely to use formal care without a significant disease diagnosis, as they have language barriers and are less aware of these services (Shrestha et al., 2023). Findings from our study suggest that these access issues should be addressed, as living with a partner or with family members appears not sufficient for managing chronic diseases and long-term health for immigrants. In fact, for some immigrant groups, family-based living arrangements may even amplify health difficulties due to factors such as cultural norms, caregiving expectations, and acculturation challenges. Therefore, it is crucial to develop comprehensive and culturally sensitive support systems that can complement and enhance the care provided within immigrant households, while also considering the cultural diversity of different immigrant communities.

### 4.3. Methodological considerations and limitations

When interpreting our findings, there are several methodological aspects and limitations to consider. First, panel attrition in SHARE may introduce selection bias, particularly if immigrants have dropped out of the survey at a higher rate than their native-born counterparts due to poor health and/or return migration to their home country. While we apply inverse probability weights based on the probability of dropping out of the panel to account for the issue of unequal attrition, it is still possible that our model is insufficient to capture all the complexities associated with the differential attrition between native-born and immigrant individuals.

Second, the potential for reverse causality challenges establishing causal relationships between living arrangements and chronic health outcomes. Changes in older adults’ living arrangements may be a response to changes in their health status. For instance, having failing health may motivate older adults to start living with family members, such as their adult children, to receive needed health support. We use lagged living arrangements in our sensitivity analyses, although endogenous relationships that extend beyond one time period may still be present.

Third, our immigrant sample presents several limitations. Most importantly, SHARE is not specifically designed to be representative of immigrant populations, likely including only those who have the legal right to stay in a European country and have sufficiently integrated to participate in a European survey. Additionally, the limited sample size prevents us from analyzing differences across receiving countries, although immigrants can have heterogeneous life experiences based on host country contexts (Kristiansen et al., 2016). Furthermore, it should be noted that our origin country groups are disproportionately represented with each of Morocco, Bosnia-Herzegovina, and Russia comprising over 20 % of the low, medium, and high HDI groups, respectively (Table S4). These sampling limitations collectively limit the generalizability of our findings to all immigrant populations in Europe.

An additional consideration relates to the self-reported doctor-diagnosed chronic conditions measure. Evidence suggests that there may be systematic differences in chronic condition reporting between immigrants and native-born individuals, potentially due to disparities in access to screening and diagnostic services and varying levels of healthcare utilization (Gimeno-Feliu et al., 2015; Solé-Auró et al., 2012). Therefore, our findings may have underestimated the true chronic disease burden of immigrants.

Finally, our approach to grouping origin countries using the HDI from 2015 presents potential limitations. Ideally, the best time point to group immigrants based on their country of origin is the year of migration, given that our aim is to determine the cultural norms and values that immigrants may have brought with them from their home countries. However, due to data availability constraints, we use the 2015 HDI, as it provides information on the greatest number of countries. While this approach is acceptable given the strong historical consistency of the HDI, some immigrants may be from countries with different development levels at the time of their migration.

## 5. Conclusion

To conclude, findings from our study provide evidence that living arrangements have different implications for age-related chronic disease accumulation among native-born and immigrant older adults in Europe. In particular, our findings suggest that among immigrants, living with a partner or with family has reduced benefits for later life health, which may be one of the mechanisms underlying their more rapid health deterioration with age. Our findings indicate that some immigrant households may have excessive caregiving burdens that prevent them from effectively managing the long-term health conditions of their older co-residents, and that might, in turn, contribute to unhealthy aging among immigrants in later life. This highlights the need to develop culturally sensitive support systems that are easily accessible for immigrant families.

## Supplementary Material

1

## Figures and Tables

**Fig. 1.**
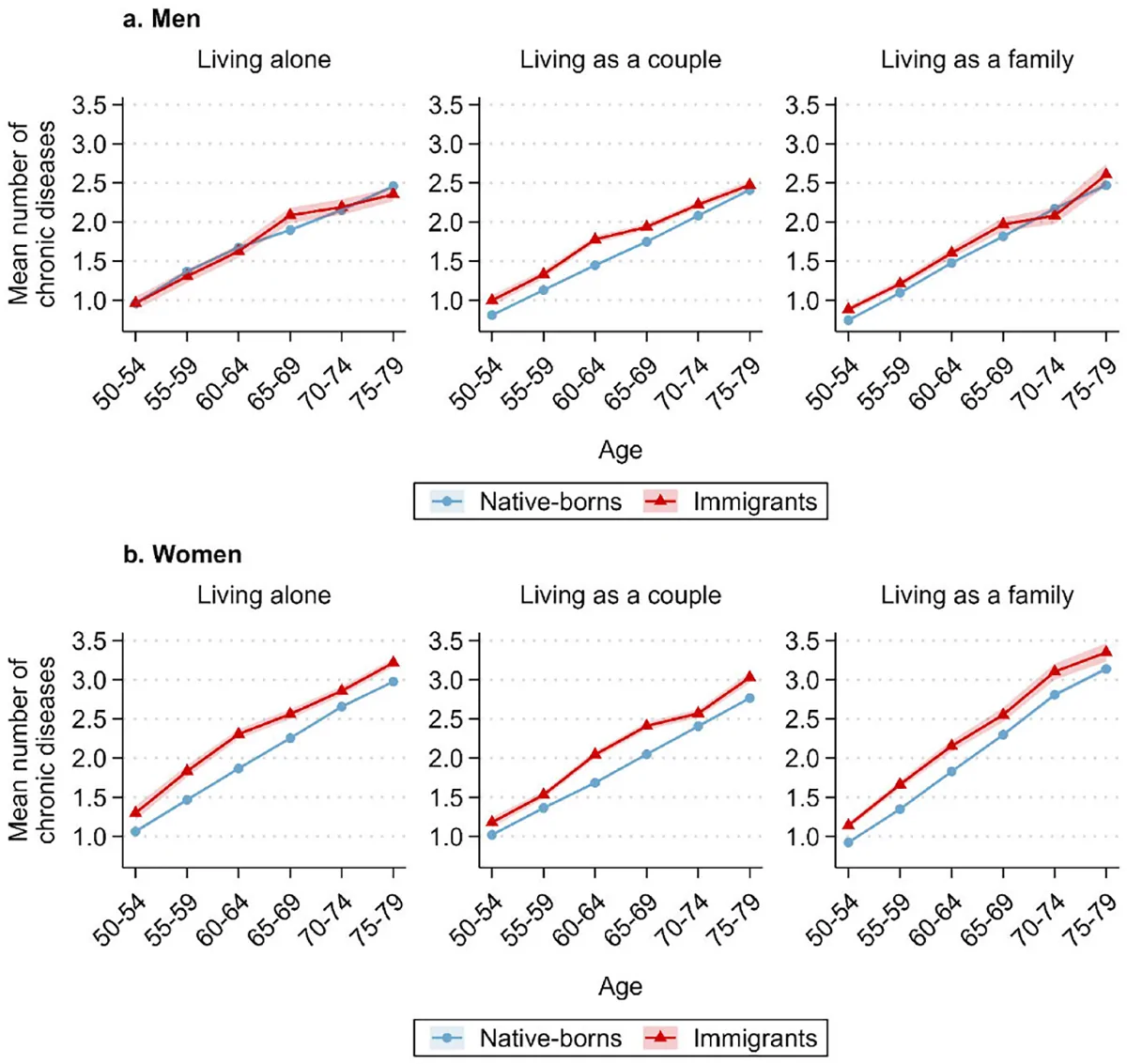
Mean number of chronic diseases in each age group of our study population by migration background and living arrangements for (a) men and (b) women.

**Fig. 2.**
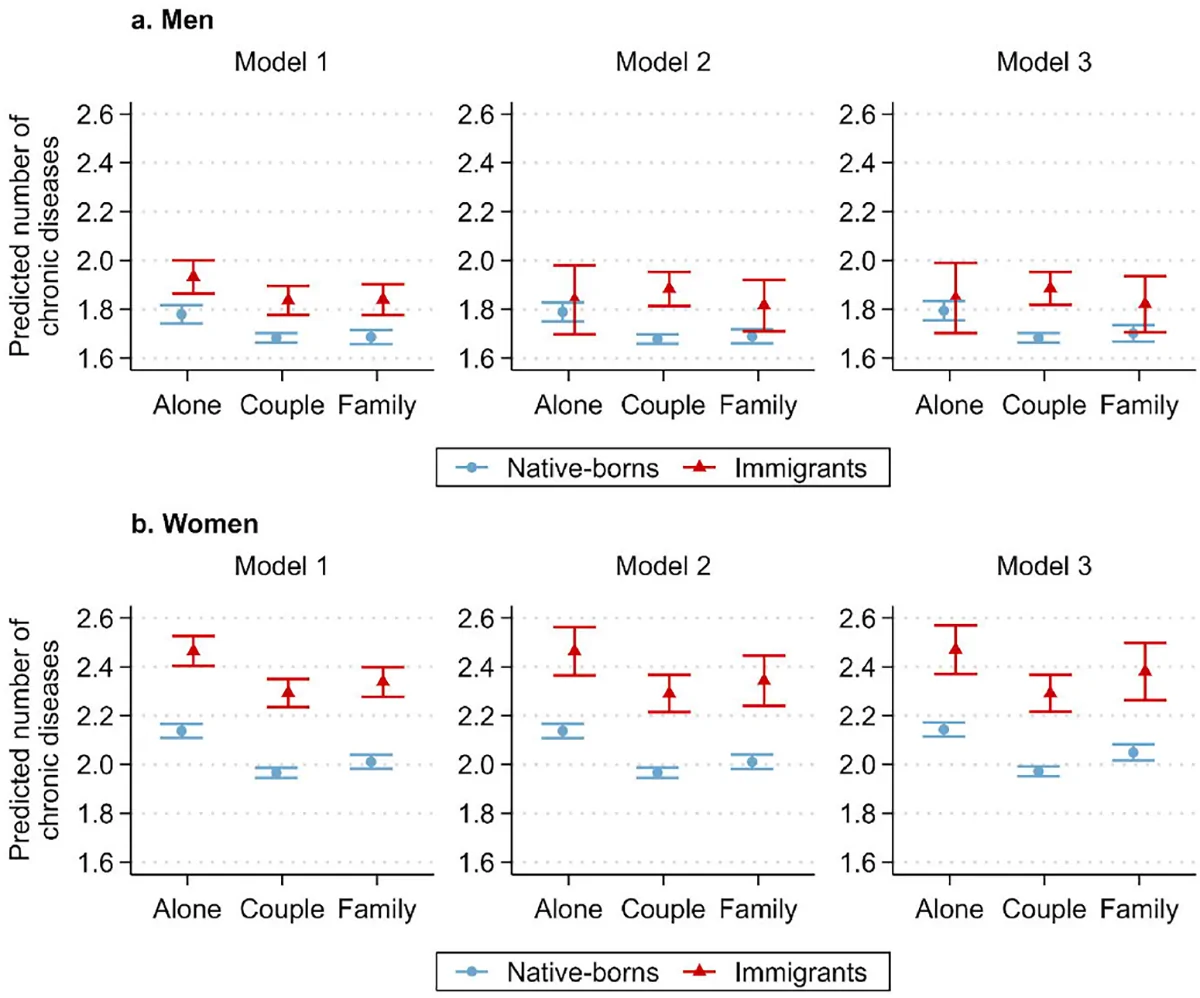
Predicted number of chronic diseases by migration background and living arrangements among (a) men and (b) women. Model 1 = migration background + age + age^2^ + migration background × age terms + living arrangements. Model 2 = Model 1 + migration background × living arrangements. Model 3 = Model 2 + migration background × age terms × living arrangements. All models are adjusted for country, survey wave, education, household income, employment status, and parental status. Non-focal predictors are set to the average level across observations in the sample.

**Fig. 3.**
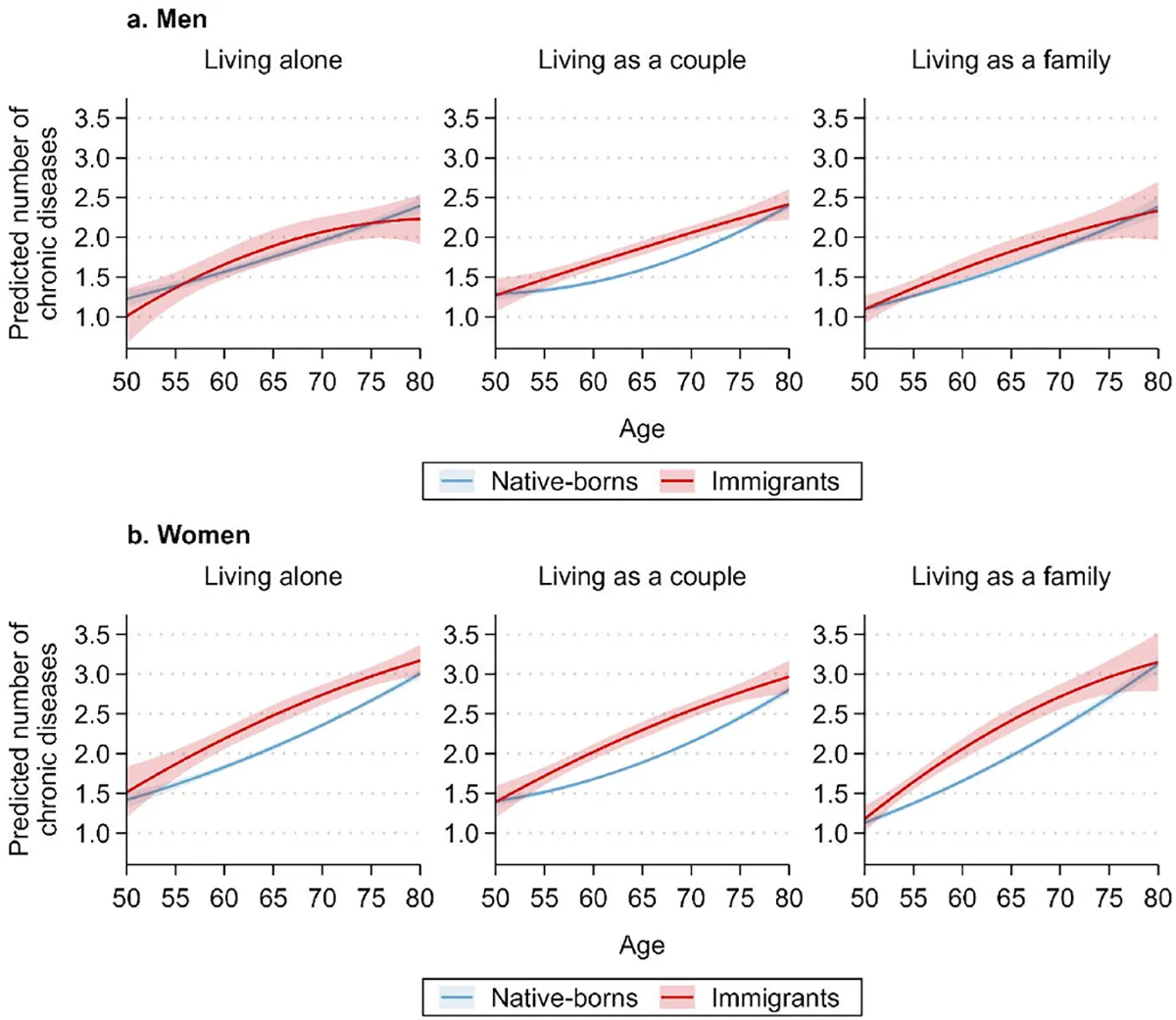
Predicted age-related profiles of chronic disease accumulation by migration background and living arrangements among (a) men and (b) women. Non-focal predictors are set to the average level across observations in the sample.

**Fig. 4.**
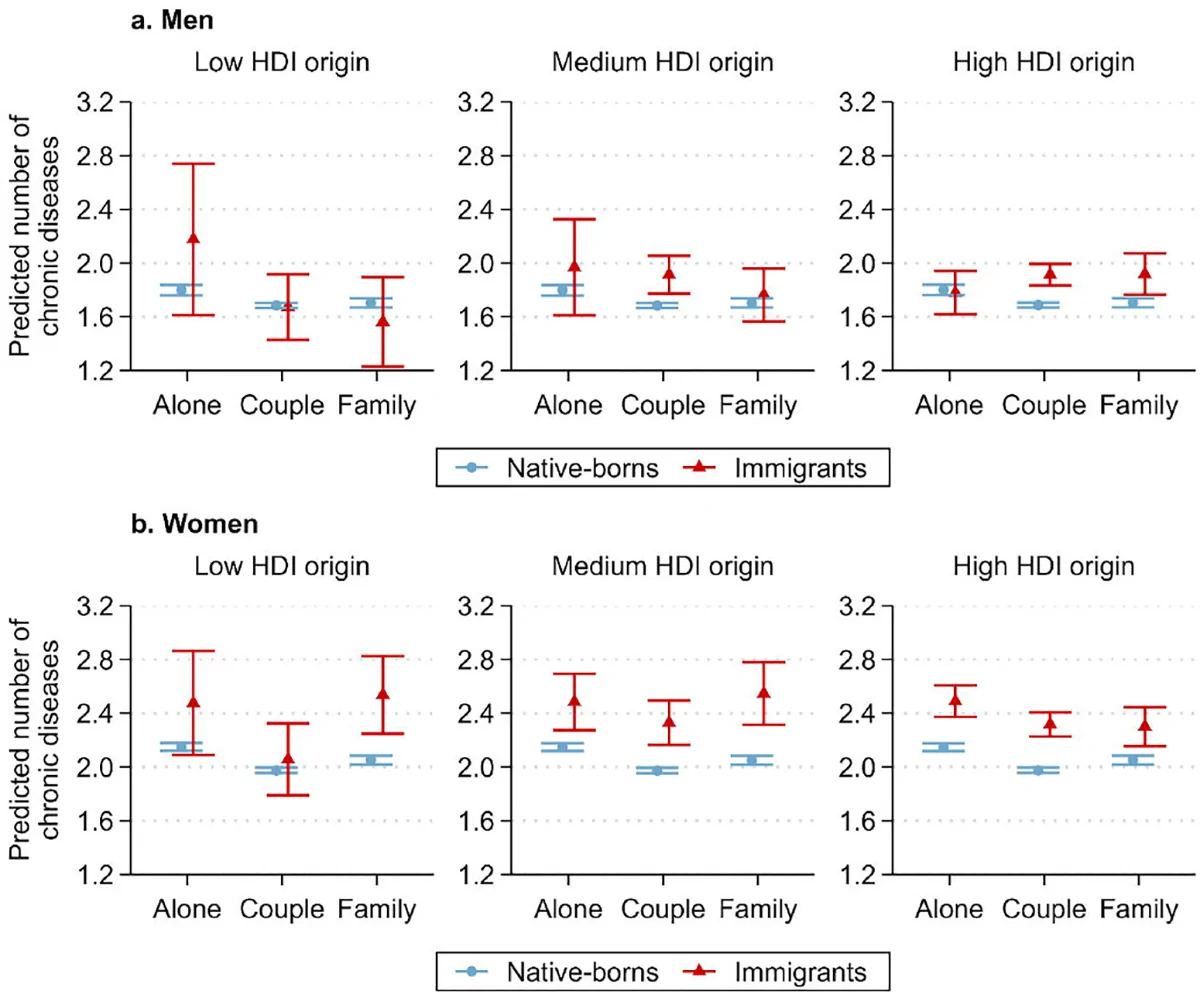
Predicted number of chronic diseases by migration background and living arrangements in samples stratified by country of origin among (a) men and (b) women. Origin countries are grouped based on the Human Development Index (HDI) into low (HDI <0.7), medium (HDI ≥0.7), and high (HDI ≥0.8) development countries. Non-focal predictors are set to the average level across observations in the sample.

**Table 1:** Model specifications.

Models	Model specification

Model 1	Migration background + Age + Migration background × Age + Living arrangements + Country + Survey wave + Confounders^a^
Model 2	Model 1 + Migration background × Living arrangements
Model 3	Model 2 + Migration background × Age × Living arrangements

a Confounders include education, household income, employment status, and parental status.

**Table 2:** Baseline characteristics^a^ of the study population by immigration background.

	Men			Women		
				
	Native-born	Immigrant		Native-born	Immigrant	

**Total**	45,623	4,318		56,876	5,541	
**Age, mean (SD)**	63.70 (8.00)	63.20 (8.23)	***	63.32 (8.23)	63.08 (8.45)	*
**Education**						
Low	14,062 (30.8%)	1,210 (28.0%)	***	21,368 (37.6%)	1,947 (35.1%)	***
Medium	20,915 (45.8%)	1,801 (41.7%)	***	23,647 (41.6%)	2,165 (39.1%)	***
High	10,646 (23.3%)	1,307 (30.3%)	***	11,861 (20.9%)	1,429 (25.8%)	***
**Household income**						
Low	15,461 (33.9%)	1,514 (35.1%)		22,475 (39.5%)	2,372 (42.8%)	***
Medium	12,690 (27.8%)	1,283 (29.7%)	**	16,010 (28.1%)	1,645 (29.7%)	*
High	17,472 (38.3%)	1,521 (35.2%)	***	18,391 (32.3%)	1,524 (27.5%)	***
**Employment status**						
Not working	29,019 (63.6%)	2,763 (64.0%)		39,281 (69.1%)	3,822 (69.0%)	
Working	16,604 (36.4%)	1,555 (36.0%)		17,595 (30.9%)	1,719 (31.0%)	
**Parental status**						
No child	9,432 (20.7%)	579 (13.4%)	***	11,172 (19.6%)	851 (15.4%)	***
Have child	36,191 (79.3%)	3,739 (86.6%)	***	45,704 (80.4%)	4,690 (84.6%)	***
**Living arrangements**						
Living alone	7,166 (15.7%)	679 (15.7%)		14,201 (25.0%)	1,526 (27.5%)	***
Live as a couple	25,360 (55.6%)	2,263 (52.4%)	***	26,611 (46.8%)	2,405 (43.4%)	***
Live as a family	13,097 (28.7%)	1,376 (31.9%)	***	16,064 (28.2%)	1,610 (29.1%)	

* p < 0.05

** p < 0.01

*** p < 0.001.

a Unweighted observations of samples at study entry.

## Data Availability

This study uses publicly available data from the Survey of Health, Ageing and Retirement in Europe (SHARE), waves 2 through 9 (https://share-eric.eu/data/).

## Data availability

The authors do not have permission to share data.

## Appendix A. Supplementary data

Supplementary data to this article can be found online at https://doi.org/10.1016/j.socscimed.2025.117954.
