# Supplementary material for: Living arrangements and chronic disease accumulation among native-born and immigrant older adults in Europe
Source: Soc Sci Med. Author manuscript; Available in PMC 2026 Oct 22. (PMC13690164; doi:10.1016/j.socscimed.2025.117954)
Supplement: Supplementary file 1 [file NIHMS2172427-supplement-1.docx]

**Online supplementary material to “Living arrangements and chronic disease accumulation among native-born and immigrant older adults in Europe”**

**Table S1.** Description of categorization for chronic health conditions

| Category | Codes of conditions/drugs from SHARE (description) |
| --- | --- |
| Heart attack/stroke | ph006d1 (a heart attack including myocardial infarction, coronary thrombosis, or any other heart problem including congestive heart failure), ph00d4 (a stroke or cerebral vascular disease), ph011d3 (drugs for other heart diseases), ph011d4 (drugs for coronary or cerebrovascular diseases) |
| Hypertension | ph006d2 (high blood pressure or hypertension), ph011d2 (drugs for high blood pressure) |
| Diabetes | ph006d5 (diabetes or high blood sugar), ph011d6(drugs for diabetes) |
| Chronic lung diseases | ph006d6 (chronic lung disease such as chronic bronchitis or emphysema), ph011d14 (drugs for chronic bronchitis) |
| Arthritis | ph006d8 (arthritis including osteoarthritis, or rheumatism; coded until 4), ph006d19 (rheumatoid arthritis; coded from wave 5 to 8), ph006d20 (osteoarthritis or other rheumatism; coded from wave 5 to 8) |
| Osteoporosis | ph011d11 (drugs for osteoporosis, hormonal; coded until wave 4), ph011d12 (drugs for osteoporosis, non-hormonal; coded until wave 4), ph011d11 (drugs for osteoporosis; coded from wave 5 to 8) |
| Mental disorders | mh022 (other affective or emotional disorders, including anxiety, nervous, or psychiatric problems; until wave 4), ph006d18 (other affective or emotional disorders including anxiety, nervous, or psychiatric problems; coded from wave 5 to 8), ph011d9 (drugs for sleep problems), ph011d10 (drugs for anxiety or depression) |
| Stomach ulcers | ph006d11 (stomach ulcer or duodenal ulcer, peptic ulcer) |
| Parkinson’s disease | ph006d12 (Parkinson’s disease) |
| Dementia | ph006d16 (Alzheimer's disease, dementia, organic brain syndrome, senility or any other serious memory impairment, organic brain syndrome, senility or any other serious memory impairment) |
| Cancer | ph006d10 (cancer or malignant tumour, including leukaemia or lymphoma, but excluding minor skin cancers) |

Table S2. Country of origin groupings by development level based on the Human Development Index (HDI) in 2015

| Low (< 0.7) | | Medium (0.700–0.799) | | High (≥ 0.800) | |
| --- | --- | --- | --- | --- | --- |
| Mean | 0.556 | Mean | 0.759 | Mean | 0.888 |
| Indonesia | 0.698 | Kazakhstan | 0.799 | Norway | 0.952 |
| Viet Nam | 0.697 | Georgia | 0.798 | Switzerland | 0.952 |
| Philippines | 0.696 | Albania | 0.797 | Iceland | 0.948 |
| Egypt | 0.695 | Serbia | 0.794 | Germany | 0.941 |
| Gabon | 0.692 | Borneo Island | 0.792 | Sweden | 0.937 |
| Kyrgyzstan | 0.689 | Costa Rica | 0.792 | Denmark | 0.936 |
| Bolivia | 0.688 | Malaysia | 0.792 | Hong Kong | 0.936 |
| Guyana | 0.686 | Mauritius | 0.791 | Singapore | 0.935 |
| El Salvador | 0.663 | Thailand | 0.789 | Australia | 0.933 |
| Cabo Verde | 0.658 | Grenada | 0.786 | New Zealand | 0.933 |
| Iraq | 0.656 | Iran | 0.782 | Netherlands | 0.932 |
| Morocco | 0.656 | North Macedonia | 0.777 | Finland | 0.930 |
| Tajikistan | 0.651 | Soviet Union | 0.775 | Canada | 0.927 |
| Equatorial Guinea | 0.648 | Mexico | 0.769 | Liechtenstein | 0.926 |
| Nicaragua | 0.644 | Armenia | 0.769 | Ireland | 0.924 |
| Bhutan | 0.625 | Venezuela | 0.766 | Belgium | 0.924 |
| India | 0.619 | Cuba | 0.765 | United States | 0.924 |
| Congo | 0.610 | Ecuador | 0.764 | United Kingdom | 0.923 |
| Honduras | 0.610 | Ukraine | 0.764 | Luxembourg | 0.914 |
| Laos | 0.604 | Sri Lanka | 0.760 | Japan | 0.913 |
| Bangladesh | 0.604 | Colombia | 0.758 | Austria | 0.910 |
| Sao Tome and Principe | 0.595 | Peru | 0.758 | Republic of Korea | 0.908 |
| Angola | 0.591 | Bosnia-Herzegovina | 0.757 | Slovenia | 0.903 |
| Ghana | 0.586 | Lebanon | 0.756 | Israel | 0.899 |
| Kenya | 0.575 | Brazil | 0.752 | France | 0.893 |
| Cambodia | 0.564 | Azerbaijan | 0.751 | Czech Republic | 0.891 |
| Zambia | 0.563 | Libya | 0.749 | Spain | 0.889 |
| Cameroon | 0.562 | Republic of Moldova | 0.749 | Malta | 0.887 |
| Comoros | 0.556 | China | 0.741 | Estonia | 0.883 |
| Syrian Arab Republic | 0.552 | Dominican Republic | 0.739 | Greece | 0.881 |
| Haiti | 0.549 | Jordan | 0.738 | Italy | 0.881 |
| Zimbabwe | 0.544 | Paraguay | 0.738 | Cyprus | 0.874 |
| Mauritania | 0.536 | Algeria | 0.736 | Czechoslovakia | 0.871 |
| Pakistan | 0.525 | Turkmenistan | 0.725 | Poland | 0.869 |
| Nigeria | 0.520 | Tunisia | 0.724 | Lithuania | 0.865 |
| Sudan | 0.514 | South Africa | 0.721 | Latvia | 0.853 |
| Togo | 0.510 | Jamaica | 0.712 | Slovakia | 0.852 |
| Benin | 0.509 | State of Palestine | 0.710 | Argentina | 0.850 |
| Rwanda | 0.509 | Suriname | 0.707 | Portugal | 0.850 |
| Tanzania | 0.507 | Uzbekistan | 0.701 | Chile | 0.846 |
| Côte d'Ivoire | 0.501 |  |  | Croatia | 0.844 |
| Senegal | 0.501 |  |  | Hungary | 0.839 |
| Madagascar | 0.499 |  |  | Montenegro | 0.827 |
| Afghanistan | 0.479 |  |  | Russian Federation | 0.823 |
| Eritrea | 0.473 |  |  | Türkiye | 0.821 |
| Liberia | 0.472 |  |  | Yugoslavia | 0.817 |
| Guinea-Bissau | 0.470 |  |  | Romania | 0.813 |
| Gambia | 0.467 |  |  | Bulgaria | 0.809 |
| Democratic Republic of  the Congo | 0.457 |  |  | Belarus | 0.809 |
| Ethiopia | 0.455 |  |  | Uruguay | 0.807 |
| Guinea | 0.449 |  |  |  |  |
| Mozambique | 0.445 |  |  |  |  |
| Sierra Leone | 0.438 |  |  |  |  |
| Burundi | 0.420 |  |  |  |  |
| Burkina Faso | 0.413 |  |  |  |  |
| Mali | 0.409 |  |  |  |  |
| Chad | 0.388 |  |  |  |  |
| Central African Republic | 0.367 |  |  |  |  |

Table S3. Country of origin groupings by national income based on gross national income (GNI) per capita in 2015

| Low (< US$ 4,035) | | Medium (US$ 4,035–12,475) | | High (> US$ 12,475) | |
| --- | --- | --- | --- | --- | --- |
| Mean | 1,729 | Mean | 7,406 | Mean | 37,244 |
| Angola | 3,930 | Türkiye | 12,080 | Norway | 93,440 |
| Sri Lanka | 3,860 | Russian Federation | 11,740 | Switzerland | 86,810 |
| Jordan | 3,860 | Kazakhstan | 11,380 | Luxembourg | 73,530 |
| State of Palestine | 3,670 | Mexico | 10,660 | Macao | 60,760 |
| El Salvador | 3,490 | Costa Rica | 10,610 | Australia | 60,550 |
| Indonesia | 3,420 | Mauritius | 10,410 | Denmark | 60,510 |
| Cabo Verde | 3,360 | Malaysia | 10,400 | Sweden | 58,440 |
| Philippines | 3,350 | Brazil | 10,160 | Faroe Islands | 57,780 |
| Republic of Moldova | 3,290 | Yugoslavia | 9,947 | United States | 56,510 |
| Morocco | 3,290 | Romania | 9,610 | Singapore | 53,160 |
| Egypt | 3,160 | Equatorial Guinea | 9,410 | Iceland | 52,790 |
| Congo | 3,100 | Libya | 8,830 | Ireland | 50,420 |
| Bolivia | 2,900 | Suriname | 8,640 | Netherlands | 49,300 |
| Nigeria | 2,860 | China | 7,890 | Canada | 47,580 |
| Ukraine | 2,800 | Gabon | 7,660 | Austria | 47,480 |
| Uzbekistan | 2,740 | Soviet Union | 7,465 | Finland | 47,180 |
| Bhutan | 2,680 | Lebanon | 7,440 | Germany | 45,780 |
| Viet Nam | 2,480 | Bulgaria | 7,430 | Belgium | 45,570 |
| Honduras | 2,020 | Colombia | 7,400 | United Kingdom | 44,380 |
| Côte d'Ivoire | 2,010 | Montenegro | 7,260 | Hong Kong | 41,180 |
| Laos | 1,970 | Cuba | 7,220 | France | 41,130 |
| Ghana | 1,870 | Grenada | 7,140 | New Zealand | 40,650 |
| Nicaragua | 1,870 | Turkmenistan | 6,790 | Japan | 39,380 |
| Mauritania | 1,690 | Belarus | 6,750 | Israel | 36,550 |
| India | 1,590 | Azerbaijan | 6,610 | Italy | 33,000 |
| Zambia | 1,540 | South Africa | 6,550 | Republic of Korea | 28,720 |
| Cameroon | 1,520 | Dominican Republic | 6,500 | Spain | 28,460 |
| Comoros | 1,470 | Peru | 6,290 | Cyprus | 26,090 |
| Haiti | 1,430 | Paraguay | 6,090 | Aruba | 25,320 |
| Sao Tome and Principe | 1,390 | Ecuador | 5,990 | Malta | 25,230 |
| Kenya | 1,330 | Serbia | 5,960 | Borneo Island | 24,325 |
| Senegal | 1,330 | Thailand | 5,580 | Slovenia | 22,270 |
| Pakistan | 1,320 | Guyana | 5,560 | Portugal | 20,460 |
| Tajikistan | 1,250 | North Macedonia | 5,550 | Greece | 20,190 |
| Sudan | 1,240 | Iran | 5,480 | Curaçao | 18,780 |
| Zimbabwe | 1,220 | Algeria | 5,460 | Estonia | 18,570 |
| Bangladesh | 1,210 | Iraq | 5,460 | Czech Republic | 18,370 |
| Kyrgyzstan | 1,180 | Bosnia-Herzegovina | 5,130 | Czechoslovakia | 18,065 |
| Benin | 1,140 | Jamaica | 4,870 | Slovakia | 17,760 |
| Cambodia | 1,070 | Georgia | 4,500 | Uruguay | 16,900 |
| Tanzania | 960 | Albania | 4,390 | Lithuania | 15,190 |
| Syrian Arab Republic | 910 | Armenia | 4,080 | Latvia | 15,110 |
| Chad | 880 | Tunisia | 4,070 | Chile | 14,220 |
| Togo | 860 |  |  | Croatia | 13,510 |
| Mali | 770 |  |  | Poland | 13,250 |
| Guinea | 750 |  |  | Hungary | 13,230 |
| Rwanda | 730 |  |  | Argentina | 12,600 |
| Burkina Faso | 680 |  |  |  |  |
| Guinea-Bissau | 670 |  |  |  |  |
| Mozambique | 660 |  |  |  |  |
| Liberia | 630 |  |  |  |  |
| Afghanistan | 600 |  |  |  |  |
| Ethiopia | 590 |  |  |  |  |
| Gambia | 580 |  |  |  |  |
| Sierra Leone | 550 |  |  |  |  |
| Somalia | 500 |  |  |  |  |
| Madagascar | 470 |  |  |  |  |
| Democratic Republic of  the Congo | 440 |  |  |  |  |
| Central African Republic | 370 |  |  |  |  |
| Burundi | 250 |  |  |  |  |

Table S4. Top five origin countries in each origin group based on the Human Development Index (HDI) and Gross National Income (GNI) per capita

| Group | Rank | By HDI | | | By GNI | | |
| --- | --- | --- | --- | --- | --- | --- | --- |
|  |  | Origin country | N | (%) | Origin country | N | (%) |
| Low |  |  |  |  |  |  |  |
|  |  | Total | 933 |  | Total | 1,231 |  |
|  | 1 | Morocco | 262 | (28.1) | Ukraine | 269 | (21.9) |
|  | 2 | Congo, both | 119 | (12.8) | Morocco | 262 | (21.3) |
|  | 3 | Indonesia | 80 | (8.6) | Congo, both | 119 | (9.7) |
|  | 4 | Egypt | 43 | (4.6) | Indonesia | 80 | (6.5) |
|  | 5 | Cabo Verde | 32 | (3.4) | Egypt | 43 | (3.5) |
| Medium |  |  |  |  |  |  |  |
|  |  | Total | 2,278 |  | Total | 4,191 |  |
|  | 1 | Bosnia-Herzegovina | 879 | (38.6) | Russia | 1,433 | (34.2) |
|  | 2 | Ukraine | 269 | (11.8) | Bosnia-Herzegovina | 879 | (21.0) |
|  | 3 | Serbia | 193 | (8.5) | Yugoslavia | 222 | (5.3) |
|  | 4 | Algeria | 189 | (8.3) | Belarus | 204 | (4.9) |
|  | 5 | Soviet Union | 88 | (3.9) | Serbia | 193 | (4.6) |
| High |  |  |  |  |  |  |  |
|  |  | Total | 6,588 |  | Total | 4,377 |  |
|  | 1 | Russia | 1,433 | (21.8) | Germany | 842 | (19.2) |
|  | 2 | Germany | 842 | (12.8) | Italy | 438 | (10.0) |
|  | 3 | Italy | 438 | (6.6) | France | 344 | (7.9) |
|  | 4 | France | 344 | (5.2) | Portugal | 293 | (6.7) |
|  | 5 | Portugal | 293 | (4.4) | Poland | 289 | (6.6) |

Table S5. Role of living arrangements in chronic disease accumulation by migration background for men and women

|  | Men | | | | | | Women | | | | | |
| --- | --- | --- | --- | --- | --- | --- | --- | --- | --- | --- | --- | --- |
|  | Model 1 | | Model 2 | | Model 3 | | Model 1 | | Model 2 | | Model 3 | |
| (Intercept) | 1.297 | *** | 1.304 | *** | 1.250 | *** | 1.150 | *** | 1.150 | *** | 1.172 | *** |
|  | (0.054) |  | (0.055) |  | (0.068) |  | (0.048) |  | (0.049) |  | (0.062) |  |
| Immigrant (ref: Native-born) | -0.077 |  | -0.148 |  | -0.218 |  | 0.044 |  | 0.040 |  | 0.096 |  |
|  | (0.069) |  | (0.097) |  | (0.186) |  | (0.065) |  | (0.087) |  | (0.171) |  |
| Age | 0.020 | *** | 0.020 | *** | 0.032 | *** | 0.038 | *** | 0.038 | *** | 0.036 | *** |
|  | (0.003) |  | (0.003) |  | (0.008) |  | (0.003) |  | (0.003) |  | (0.006) |  |
| Age^2^ | 0.001 | *** | 0.001 | *** | 0.000 |  | 0.001 | *** | 0.001 | *** | 0.001 | *** |
|  | (0.000) |  | (0.000) |  | (0.000) |  | (0.000) |  | (0.000) |  | (0.000) |  |
| Immigrant × age | 0.039 | *** | 0.035 | ** | 0.046 |  | 0.046 | *** | 0.046 | *** | 0.038 |  |
|  | (0.011) |  | (0.011) |  | (0.028) |  | (0.010) |  | (0.011) |  | (0.022) |  |
| Immigrant × age^2^ | -0.001 | *** | -0.001 | ** | -0.001 |  | -0.001 | *** | -0.001 | *** | -0.001 |  |
|  | (0.000) |  | (0.000) |  | (0.001) |  | (0.000) |  | (0.000) |  | (0.001) |  |
| Living arrangements (ref: Alone) |  |  |  |  |  |  |  |  |  |  |  |  |
| Living as a couple | -0.097 | *** | -0.111 | *** | 0.064 |  | -0.171 | *** | -0.171 | *** | -0.016 |  |
|  | (0.022) |  | (0.022) |  | (0.060) |  | (0.018) |  | (0.018) |  | (0.054) |  |
| Living as a family | -0.093 | *** | -0.100 | *** | -0.127 | * | -0.126 | *** | -0.127 | *** | -0.287 | *** |
|  | (0.024) |  | (0.025) |  | (0.057) |  | (0.021) |  | (0.021) |  | (0.052) |  |
| Immigrant × couple |  |  | 0.156 |  | 0.197 |  |  |  | -0.002 |  | -0.108 |  |
|  |  |  | (0.082) |  | (0.216) |  |  |  | (0.064) |  | (0.200) |  |
| Immigrant × family |  |  | 0.076 |  | 0.211 |  |  |  | 0.006 |  | -0.051 |  |
|  |  |  | (0.089) |  | (0.205) |  |  |  | (0.074) |  | (0.186) |  |
| Native-born × age × couple |  |  |  |  | -0.028 | ** |  |  |  |  | -0.017 | * |
|  |  |  |  |  | (0.009) |  |  |  |  |  | (0.007) |  |
| Native-born × age × family |  |  |  |  | -0.001 |  |  |  |  |  | 0.010 |  |
|  |  |  |  |  | (0.009) |  |  |  |  |  | (0.008) |  |
| Native-born × age^2^ × couple |  |  |  |  | 0.001 | ** |  |  |  |  | 0.000 |  |
|  |  |  |  |  | (0.000) |  |  |  |  |  | (0.000) |  |
| Native-born × age^2^ × family |  |  |  |  | 0.000 |  |  |  |  |  | 0.000 |  |
|  |  |  |  |  | (0.000) |  |  |  |  |  | (0.000) |  |
| Immigrant × age × couple |  |  |  |  | -0.036 |  |  |  |  |  | -0.005 |  |
|  |  |  |  |  | (0.031) |  |  |  |  |  | (0.026) |  |
| Immigrant × age × family |  |  |  |  | -0.021 |  |  |  |  |  | 0.026 |  |
|  |  |  |  |  | (0.032) |  |  |  |  |  | (0.027) |  |
| Immigrant × age^2^ × couple |  |  |  |  | 0.001 |  |  |  |  |  | 0.000 |  |
|  |  |  |  |  | (0.001) |  |  |  |  |  | (0.001) |  |
| Immigrant × age^2^ × family |  |  |  |  | 0.001 |  |  |  |  |  | -0.001 |  |
|  |  |  |  |  | (0.001) |  |  |  |  |  | (0.001) |  |
| Education (ref: Low) |  |  |  |  |  |  |  |  |  |  |  |  |
| Medium | -0.189 | *** | -0.189 | *** | -0.188 | *** | -0.287 | *** | -0.287 | *** | -0.281 | *** |
|  | (0.021) |  | (0.021) |  | (0.021) |  | (0.020) |  | (0.020) |  | (0.020) |  |
| High | -0.355 | *** | -0.354 | *** | -0.352 | *** | -0.508 | *** | -0.508 | *** | -0.499 | *** |
|  | (0.023) |  | (0.023) |  | (0.023) |  | (0.022) |  | (0.022) |  | (0.022) |  |
| Household income (ref: Low) |  |  |  |  |  |  |  |  |  |  |  |  |
| Medium | -0.025 |  | -0.026 |  | -0.024 |  | -0.070 | *** | -0.070 | *** | -0.059 | *** |
|  | (0.015) |  | (0.015) |  | (0.015) |  | (0.014) |  | (0.014) |  | (0.014) |  |
| High | -0.128 | *** | -0.129 | *** | -0.127 | *** | -0.150 | *** | -0.150 | *** | -0.145 | *** |
|  | (0.015) |  | (0.015) |  | (0.015) |  | (0.014) |  | (0.014) |  | (0.014) |  |
| Working (ref: Not working) | -0.570 | *** | -0.570 | *** | -0.574 | *** | -0.512 | *** | -0.512 | *** | -0.523 | *** |
|  | (0.018) |  | (0.018) |  | (0.018) |  | (0.017) |  | (0.017) |  | (0.017) |  |
| Have child (ref: No child) | 0.041 |  | 0.043 |  | 0.047 | * | 0.123 | *** | 0.123 | *** | 0.127 | *** |
|  | (0.023) |  | (0.022) |  | (0.022) |  | (0.021) |  | (0.021) |  | (0.021) |  |
| Country (ref: Austria) |  |  |  |  |  |  |  |  |  |  |  |  |
| Belgium | 0.270 | *** | 0.269 | *** | 0.272 | *** | 0.622 | *** | 0.622 | *** | 0.629 | *** |
|  | (0.047) |  | (0.047) |  | (0.047) |  | (0.046) |  | (0.046) |  | (0.046) |  |
| Bulgaria | -0.339 | *** | -0.340 | *** | -0.341 | *** | -0.091 |  | -0.091 |  | -0.100 |  |
|  | (0.067) |  | (0.067) |  | (0.067) |  | (0.060) |  | (0.060) |  | (0.060) |  |
| Croatia | -0.283 | *** | -0.284 | *** | -0.284 | *** | -0.047 |  | -0.047 |  | -0.047 |  |
|  | (0.050) |  | (0.050) |  | (0.050) |  | (0.049) |  | (0.049) |  | (0.049) |  |
| Cyprus | -0.463 | *** | -0.463 | *** | -0.460 | *** | -0.262 | *** | -0.262 | *** | -0.244 | ** |
|  | (0.079) |  | (0.079) |  | (0.079) |  | (0.074) |  | (0.074) |  | (0.074) |  |
| Czech Republic | 0.285 | *** | 0.284 | *** | 0.285 | *** | 0.352 | *** | 0.352 | *** | 0.352 | *** |
|  | (0.051) |  | (0.051) |  | (0.051) |  | (0.045) |  | (0.045) |  | (0.045) |  |
| Denmark | 0.078 |  | 0.078 |  | 0.080 |  | 0.130 | ** | 0.130 | ** | 0.132 | ** |
|  | (0.050) |  | (0.050) |  | (0.050) |  | (0.047) |  | (0.047) |  | (0.047) |  |
| Estonia | 0.309 | *** | 0.308 | *** | 0.308 | *** | 0.485 | *** | 0.485 | *** | 0.483 | *** |
|  | (0.050) |  | (0.050) |  | (0.050) |  | (0.045) |  | (0.045) |  | (0.045) |  |
| Finland | -0.243 | *** | -0.243 | *** | -0.242 | *** | -0.242 | *** | -0.242 | *** | -0.235 | *** |
|  | (0.059) |  | (0.059) |  | (0.059) |  | (0.056) |  | (0.056) |  | (0.056) |  |
| France | 0.040 |  | 0.039 |  | 0.042 |  | 0.340 | *** | 0.340 | *** | 0.344 | *** |
|  | (0.048) |  | (0.048) |  | (0.048) |  | (0.046) |  | (0.046) |  | (0.046) |  |
| Germany | 0.175 | *** | 0.175 | *** | 0.176 | *** | 0.156 | *** | 0.156 | *** | 0.159 | *** |
|  | (0.047) |  | (0.047) |  | (0.047) |  | (0.044) |  | (0.044) |  | (0.044) |  |
| Greece | -0.422 | *** | -0.424 | *** | -0.425 | *** | -0.179 | *** | -0.179 | *** | -0.176 | *** |
|  | (0.047) |  | (0.047) |  | (0.047) |  | (0.046) |  | (0.046) |  | (0.046) |  |
| Hungary | 0.297 | *** | 0.297 | *** | 0.297 | *** | 0.566 | *** | 0.566 | *** | 0.562 | *** |
|  | (0.058) |  | (0.058) |  | (0.058) |  | (0.056) |  | (0.056) |  | (0.056) |  |
| Ireland | -0.102 |  | -0.105 |  | -0.102 |  | -0.166 | * | -0.166 | * | -0.164 | * |
|  | (0.076) |  | (0.076) |  | (0.076) |  | (0.073) |  | (0.073) |  | (0.074) |  |
| Italy | -0.007 |  | -0.008 |  | -0.007 |  | 0.219 | *** | 0.219 | *** | 0.227 | *** |
|  | (0.050) |  | (0.050) |  | (0.050) |  | (0.047) |  | (0.047) |  | (0.047) |  |
| Latvia | -0.439 | *** | -0.441 | *** | -0.443 | *** | -0.144 | ** | -0.144 | ** | -0.151 | ** |
|  | (0.058) |  | (0.058) |  | (0.058) |  | (0.055) |  | (0.055) |  | (0.055) |  |
| Lithuania | -0.005 |  | -0.007 |  | -0.008 |  | 0.368 | *** | 0.368 | *** | 0.360 | *** |
|  | (0.073) |  | (0.073) |  | (0.073) |  | (0.060) |  | (0.060) |  | (0.060) |  |
| Luxembourg | 0.043 |  | 0.040 |  | 0.043 |  | 0.139 | * | 0.139 | * | 0.152 | * |
|  | (0.069) |  | (0.069) |  | (0.069) |  | (0.069) |  | (0.069) |  | (0.069) |  |
| Malta | -0.492 | *** | -0.493 | *** | -0.494 | *** | -0.521 | *** | -0.520 | *** | -0.517 | *** |
|  | (0.077) |  | (0.077) |  | (0.077) |  | (0.067) |  | (0.067) |  | (0.067) |  |
| Netherlands | -0.342 | *** | -0.341 | *** | -0.336 | *** | -0.276 | *** | -0.276 | *** | -0.264 | *** |
|  | (0.049) |  | (0.049) |  | (0.049) |  | (0.047) |  | (0.047) |  | (0.047) |  |
| Poland | 0.147 | ** | 0.146 | ** | 0.146 | ** | 0.375 | *** | 0.375 | *** | 0.368 | *** |
|  | (0.050) |  | (0.050) |  | (0.050) |  | (0.047) |  | (0.047) |  | (0.047) |  |
| Portugal | 0.141 | * | 0.141 | * | 0.141 | * | 0.723 | *** | 0.723 | *** | 0.725 | *** |
|  | (0.068) |  | (0.068) |  | (0.068) |  | (0.065) |  | (0.065) |  | (0.064) |  |
| Romania | -0.533 | *** | -0.533 | *** | -0.534 | *** | -0.080 |  | -0.080 |  | -0.095 |  |
|  | (0.063) |  | (0.063) |  | (0.063) |  | (0.060) |  | (0.060) |  | (0.060) |  |
| Slovakia | -0.661 | *** | -0.661 | *** | -0.658 | *** | -0.242 | *** | -0.242 | *** | -0.241 | *** |
|  | (0.058) |  | (0.058) |  | (0.058) |  | (0.065) |  | (0.065) |  | (0.065) |  |
| Slovenia | -0.156 | ** | -0.159 | ** | -0.159 | ** | -0.094 | * | -0.094 | * | -0.095 | * |
|  | (0.050) |  | (0.050) |  | (0.050) |  | (0.046) |  | (0.046) |  | (0.046) |  |
| Spain | -0.009 |  | -0.010 |  | -0.010 |  | 0.313 | *** | 0.313 | *** | 0.314 | *** |
|  | (0.051) |  | (0.051) |  | (0.051) |  | (0.049) |  | (0.049) |  | (0.049) |  |
| Sweden | -0.237 | *** | -0.238 | *** | -0.234 | *** | -0.113 | * | -0.113 | * | -0.100 | * |
|  | (0.051) |  | (0.051) |  | (0.051) |  | (0.049) |  | (0.049) |  | (0.049) |  |
| Switzerland | -0.213 | *** | -0.213 | *** | -0.210 | *** | -0.230 | *** | -0.230 | *** | -0.220 | *** |
|  | (0.053) |  | (0.053) |  | (0.053) |  | (0.051) |  | (0.051) |  | (0.051) |  |
| Wave (ref: Wave 2) |  |  |  |  |  |  |  |  |  |  |  |  |
| Wave 4 | 0.063 | *** | 0.063 | *** | 0.062 | *** | 0.061 | *** | 0.061 | *** | 0.061 | *** |
|  | (0.015) |  | (0.015) |  | (0.015) |  | (0.014) |  | (0.014) |  | (0.014) |  |
| Wave 5 | 0.202 | *** | 0.202 | *** | 0.201 | *** | 0.220 | *** | 0.220 | *** | 0.220 | *** |
|  | (0.015) |  | (0.015) |  | (0.015) |  | (0.015) |  | (0.015) |  | (0.015) |  |
| Wave 6 | 0.338 | *** | 0.338 | *** | 0.337 | *** | 0.390 | *** | 0.390 | *** | 0.390 | *** |
|  | (0.016) |  | (0.016) |  | (0.016) |  | (0.016) |  | (0.016) |  | (0.016) |  |
| Wave 7 | 0.473 | *** | 0.474 | *** | 0.475 | *** | 0.573 | *** | 0.573 | *** | 0.581 | *** |
|  | (0.022) |  | (0.022) |  | (0.022) |  | (0.021) |  | (0.021) |  | (0.021) |  |
| Wave 8 | 0.512 | *** | 0.512 | *** | 0.511 | *** | 0.536 | *** | 0.536 | *** | 0.538 | *** |
|  | (0.018) |  | (0.018) |  | (0.018) |  | (0.018) |  | (0.018) |  | (0.018) |  |
| Wave 9 | 0.567 | *** | 0.567 | *** | 0.565 | *** | 0.560 | *** | 0.560 | *** | 0.563 | *** |
|  | (0.018) |  | (0.018) |  | (0.018) |  | (0.018) |  | (0.018) |  | (0.018) |  |
| Number of persons | 49,941 |  | 49,941 |  | 49,941 |  | 62,417 |  | 62,417 |  | 62,417 |  |
| Number of observations | 112,132 |  | 112,132 |  | 112,132 |  | 151,690 |  | 151,690 |  | 151,690 |  |
| R-squared | 0.180 |  | 0.180 |  | 0.181 |  | 0.213 |  | 0.213 |  | 0.214 |  |
| Inverse probability weights | 1.647 |  | 1.647 |  | 1.647 |  | 1.532 |  | 1.532 |  | 1.532 |  |
|  | (0.002) |  | (0.002) |  | (0.002) |  | (0.001) |  | (0.001) |  | (0.001) |  |

* p < 0.05; ** p < 0.01; *** p < 0.001

Table S6. Predicted number of chronic diseases for men and women by migration background and living arrangements

|  | Men | | Women | |
| --- | --- | --- | --- | --- |
|  | Native-born | Immigrant | Native-born | Immigrant |
| Model 1 |  |  |  |  |
| Living alone | 1.78 (1.74, 1.82) | 1.93 (1.87, 2.00) | 2.14 (2.11, 2.17) | 2.46 (2.40, 2.53) |
| Living as a couple | 1.68 (1.66, 1.70) | 1.84 (1.78, 1.90) | 1.97 (1.95, 1.99) | 2.29 (2.24, 2.35) |
| Living as a family | 1.69 (1.66, 1.72) | 1.84 (1.78, 1.90) | 2.01 (1.98, 2.04) | 2.34 (2.28, 2.40) |
| Model 2 |  |  |  |  |
| Living alone | 1.79 (1.75, 1.83) | 1.84 (1.70, 1.98) | 2.14 (2.11, 2.17) | 2.46 (2.37, 2.56) |
| Living as a couple | 1.68 (1.66, 1.70) | 1.88 (1.81, 1.95) | 1.97 (1.95, 1.99) | 2.29 (2.21, 2.37) |
| Living as a family | 1.69 (1.66, 1.72) | 1.82 (1.71, 1.92) | 2.01 (1.98, 2.04) | 2.34 (2.24, 2.45) |
| Model 3 |  |  |  |  |
| Living alone | 1.79 (1.76, 1.83) | 1.85 (1.70, 1.99) | 2.14 (2.11, 2.17) | 2.47 (2.37, 2.57) |
| Living as a couple | 1.68 (1.66, 1.70) | 1.89 (1.82, 1.95) | 1.97 (1.95, 1.99) | 2.29 (2.22, 2.37) |
| Living as a family | 1.70 (1.67, 1.74) | 1.82 (1.71, 1.94) | 2.05 (2.02, 2.08) | 2.38 (2.26, 2.50) |

Table S7. Role of living arrangements in chronic disease accumulation by migration background for men and women, with immigrant samples stratified by the Human Development Index (HDI) of the origin country

|  | Men | | | | | | Women | | | | | |
| --- | --- | --- | --- | --- | --- | --- | --- | --- | --- | --- | --- | --- |
|  | Low | | Medium | | High | | Low | | Medium | | High | |
| (Intercept) | 1.223 | *** | 1.228 | *** | 1.241 | *** | 1.160 | *** | 1.173 | *** | 1.170 | *** |
|  | (0.069) |  | (0.069) |  | (0.068) |  | (0.063) |  | (0.063) |  | (0.063) |  |
| Immigrant (ref: Native-born) | -0.009 |  | -0.517 |  | -0.264 |  | -0.496 |  | -0.070 |  | 0.236 |  |
|  | (0.569) |  | (0.414) |  | (0.192) |  | (0.561) |  | (0.310) |  | (0.209) |  |
| Age | 0.033 | *** | 0.033 | *** | 0.032 | *** | 0.037 | *** | 0.036 | *** | 0.036 | *** |
|  | (0.008) |  | (0.008) |  | (0.008) |  | (0.006) |  | (0.006) |  | (0.006) |  |
| Age^2^ | 0.000 |  | 0.000 |  | 0.000 |  | 0.001 | ** | 0.001 | ** | 0.001 | ** |
|  | (0.000) |  | (0.000) |  | (0.000) |  | (0.000) |  | (0.000) |  | (0.000) |  |
| Immigrant × age | 0.099 |  | 0.087 |  | 0.038 |  | 0.120 |  | 0.053 |  | 0.023 |  |
|  | (0.093) |  | (0.069) |  | (0.030) |  | (0.075) |  | (0.042) |  | (0.026) |  |
| Immigrant × age^2^ | -0.004 |  | -0.002 |  | -0.001 |  | -0.003 |  | -0.001 |  | -0.001 |  |
|  | (0.003) |  | (0.002) |  | (0.001) |  | (0.002) |  | (0.001) |  | (0.001) |  |
| Living arrangements (ref: Alone) |  |  |  |  |  |  |  |  |  |  |  |  |
| Living as a couple | 0.055 |  | 0.055 |  | 0.062 |  | -0.019 |  | -0.020 |  | -0.015 |  |
|  | (0.060) |  | (0.060) |  | (0.060) |  | (0.054) |  | (0.054) |  | (0.054) |  |
| Living as a family | -0.136 | * | -0.134 | * | -0.131 | * | -0.295 | *** | -0.296 | *** | -0.285 | *** |
|  | (0.057) |  | (0.057) |  | (0.057) |  | (0.052) |  | (0.052) |  | (0.052) |  |
| Immigrant × couple | -0.272 |  | 0.507 |  | 0.310 |  | 0.008 |  | 0.138 |  | -0.206 |  |
|  | (0.638) |  | (0.465) |  | (0.236) |  | (0.658) |  | (0.396) |  | (0.241) |  |
| Immigrant × family | 0.053 |  | 0.391 |  | 0.283 |  | 0.684 |  | -0.017 |  | -0.159 |  |
|  | (0.619) |  | (0.428) |  | (0.228) |  | (0.563) |  | (0.335) |  | (0.232) |  |
| Native-born × age × couple | -0.027 | ** | -0.027 | ** | -0.028 | ** | -0.017 | * | -0.017 | * | -0.017 | * |
|  | (0.009) |  | (0.009) |  | (0.009) |  | (0.007) |  | (0.007) |  | (0.007) |  |
| Native-born × age × family | -0.000 |  | -0.000 |  | -0.001 |  | 0.011 |  | 0.011 |  | 0.010 |  |
|  | (0.009) |  | (0.009) |  | (0.009) |  | (0.008) |  | (0.008) |  | (0.008) |  |
| Native-born × age^2^ × couple | 0.001 | ** | 0.001 | ** | 0.001 | ** | 0.000 |  | 0.000 |  | 0.000 |  |
|  | (0.000) |  | (0.000) |  | (0.000) |  | (0.000) |  | (0.000) |  | (0.000) |  |
| Native-born × age^2^ × family | 0.000 |  | 0.000 |  | 0.000 |  | 0.000 |  | 0.000 |  | 0.000 |  |
|  | (0.000) |  | (0.000) |  | (0.000) |  | (0.000) |  | (0.000) |  | (0.000) |  |
| Immigrant × age × couple | -0.093 |  | -0.082 |  | -0.030 |  | -0.024 |  | -0.060 |  | 0.011 |  |
|  | (0.103) |  | (0.075) |  | (0.034) |  | (0.091) |  | (0.054) |  | (0.031) |  |
| Immigrant × age × family | -0.084 |  | -0.062 |  | -0.008 |  | -0.109 |  | 0.071 |  | 0.022 |  |
|  | (0.106) |  | (0.074) |  | (0.038) |  | (0.082) |  | (0.054) |  | (0.034) |  |
| Immigrant × age^2^ × couple | 0.004 |  | 0.002 |  | 0.001 |  | -0.000 |  | 0.002 |  | -0.000 |  |
|  | (0.004) |  | (0.002) |  | (0.001) |  | (0.003) |  | (0.002) |  | (0.001) |  |
| Immigrant × age^2^ × family | 0.003 |  | 0.002 |  | 0.000 |  | 0.005 |  | -0.002 |  | -0.000 |  |
|  | (0.004) |  | (0.002) |  | (0.001) |  | (0.003) |  | (0.002) |  | (0.001) |  |
| Education (ref: Low) |  |  |  |  |  |  |  |  |  |  |  |  |
| Medium | -0.190 | *** | -0.191 | *** | -0.192 | *** | -0.279 | *** | -0.284 | *** | -0.282 | *** |
|  | (0.022) |  | (0.022) |  | (0.021) |  | (0.020) |  | (0.020) |  | (0.020) |  |
| High | -0.342 | *** | -0.349 | *** | -0.354 | *** | -0.488 | *** | -0.495 | *** | -0.503 | *** |
|  | (0.024) |  | (0.024) |  | (0.024) |  | (0.023) |  | (0.023) |  | (0.023) |  |
| Household income (ref: Low) |  |  |  |  |  |  |  |  |  |  |  |  |
| Medium | -0.022 |  | -0.023 |  | -0.021 |  | -0.049 | *** | -0.052 | *** | -0.058 | *** |
|  | (0.016) |  | (0.016) |  | (0.016) |  | (0.014) |  | (0.014) |  | (0.014) |  |
| High | -0.122 | *** | -0.122 | *** | -0.127 | *** | -0.137 | *** | -0.139 | *** | -0.145 | *** |
|  | (0.016) |  | (0.015) |  | (0.015) |  | (0.014) |  | (0.014) |  | (0.014) |  |
| Working (ref: Not working) | -0.554 | *** | -0.555 | *** | -0.571 | *** | -0.508 | *** | -0.512 | *** | -0.514 | *** |
|  | (0.018) |  | (0.018) |  | (0.018) |  | (0.017) |  | (0.017) |  | (0.017) |  |
| Have child (ref: No child) | 0.043 |  | 0.044 |  | 0.052 | * | 0.130 | *** | 0.136 | *** | 0.124 | *** |
|  | (0.023) |  | (0.023) |  | (0.023) |  | (0.022) |  | (0.022) |  | (0.022) |  |
| Country (ref: Austria) |  |  |  |  |  |  |  |  |  |  |  |  |
| Belgium | 0.298 | *** | 0.291 | *** | 0.274 | *** | 0.645 | *** | 0.636 | *** | 0.631 | *** |
|  | (0.050) |  | (0.049) |  | (0.048) |  | (0.047) |  | (0.047) |  | (0.046) |  |
| Bulgaria | -0.319 | *** | -0.325 | *** | -0.333 | *** | -0.099 |  | -0.107 |  | -0.101 |  |
|  | (0.068) |  | (0.068) |  | (0.067) |  | (0.061) |  | (0.061) |  | (0.061) |  |
| Croatia | -0.221 | *** | -0.264 | *** | -0.240 | *** | 0.008 |  | -0.053 |  | -0.008 |  |
|  | (0.052) |  | (0.051) |  | (0.051) |  | (0.052) |  | (0.050) |  | (0.051) |  |
| Cyprus | -0.457 | *** | -0.455 | *** | -0.460 | *** | -0.234 | ** | -0.239 | ** | -0.252 | *** |
|  | (0.080) |  | (0.079) |  | (0.079) |  | (0.076) |  | (0.076) |  | (0.075) |  |
| Czech Republic | 0.297 | *** | 0.293 | *** | 0.288 | *** | 0.355 | *** | 0.348 | *** | 0.348 | *** |
|  | (0.052) |  | (0.052) |  | (0.051) |  | (0.046) |  | (0.046) |  | (0.045) |  |
| Denmark | 0.090 |  | 0.090 |  | 0.083 |  | 0.120 | * | 0.116 | * | 0.126 | ** |
|  | (0.052) |  | (0.052) |  | (0.050) |  | (0.048) |  | (0.048) |  | (0.048) |  |
| Estonia | 0.294 | *** | 0.303 | *** | 0.297 | *** | 0.396 | *** | 0.403 | *** | 0.472 | *** |
|  | (0.053) |  | (0.053) |  | (0.051) |  | (0.048) |  | (0.047) |  | (0.046) |  |
| Finland | -0.228 | *** | -0.233 | *** | -0.236 | *** | -0.239 | *** | -0.245 | *** | -0.237 | *** |
|  | (0.060) |  | (0.060) |  | (0.059) |  | (0.057) |  | (0.057) |  | (0.056) |  |
| France | 0.052 |  | 0.045 |  | 0.054 |  | 0.342 | *** | 0.334 | *** | 0.335 | *** |
|  | (0.050) |  | (0.050) |  | (0.049) |  | (0.047) |  | (0.047) |  | (0.046) |  |
| Germany | 0.211 | *** | 0.208 | *** | 0.189 | *** | 0.156 | *** | 0.151 | *** | 0.159 | *** |
|  | (0.049) |  | (0.049) |  | (0.047) |  | (0.045) |  | (0.045) |  | (0.044) |  |
| Greece | -0.412 | *** | -0.422 | *** | -0.421 | *** | -0.178 | *** | -0.188 | *** | -0.177 | *** |
|  | (0.048) |  | (0.048) |  | (0.047) |  | (0.047) |  | (0.047) |  | (0.047) |  |
| Hungary | 0.306 | *** | 0.302 | *** | 0.303 | *** | 0.562 | *** | 0.552 | *** | 0.564 | *** |
|  | (0.059) |  | (0.059) |  | (0.058) |  | (0.056) |  | (0.056) |  | (0.056) |  |
| Ireland | -0.095 |  | -0.090 |  | -0.094 |  | -0.196 | ** | -0.201 | ** | -0.166 | * |
|  | (0.080) |  | (0.080) |  | (0.077) |  | (0.075) |  | (0.075) |  | (0.074) |  |
| Italy | 0.002 |  | -0.002 |  | -0.002 |  | 0.231 | *** | 0.224 | *** | 0.224 | *** |
|  | (0.051) |  | (0.051) |  | (0.050) |  | (0.048) |  | (0.048) |  | (0.047) |  |
| Latvia | -0.411 | *** | -0.411 | *** | -0.444 | *** | -0.117 | * | -0.122 | * | -0.156 | ** |
|  | (0.062) |  | (0.061) |  | (0.059) |  | (0.057) |  | (0.056) |  | (0.056) |  |
| Lithuania | -0.008 |  | -0.011 |  | -0.006 |  | 0.350 | *** | 0.351 | *** | 0.352 | *** |
|  | (0.075) |  | (0.075) |  | (0.073) |  | (0.062) |  | (0.061) |  | (0.061) |  |
| Luxembourg | 0.119 |  | 0.123 |  | 0.055 |  | 0.234 | ** | 0.198 | * | 0.127 |  |
|  | (0.083) |  | (0.084) |  | (0.071) |  | (0.080) |  | (0.080) |  | (0.070) |  |
| Malta | -0.490 | *** | -0.499 | *** | -0.487 | *** | -0.509 | *** | -0.518 | *** | -0.518 | *** |
|  | (0.075) |  | (0.075) |  | (0.077) |  | (0.068) |  | (0.068) |  | (0.067) |  |
| Netherlands | -0.334 | *** | -0.340 | *** | -0.329 | *** | -0.269 | *** | -0.282 | *** | -0.270 | *** |
|  | (0.050) |  | (0.050) |  | (0.049) |  | (0.048) |  | (0.048) |  | (0.047) |  |
| Poland | 0.162 | ** | 0.159 | ** | 0.150 | ** | 0.370 | *** | 0.361 | *** | 0.368 | *** |
|  | (0.052) |  | (0.051) |  | (0.050) |  | (0.048) |  | (0.048) |  | (0.047) |  |
| Portugal | 0.165 | * | 0.152 | * | 0.149 | * | 0.730 | *** | 0.737 | *** | 0.742 | *** |
|  | (0.069) |  | (0.070) |  | (0.069) |  | (0.065) |  | (0.066) |  | (0.066) |  |
| Romania | -0.508 | *** | -0.516 | *** | -0.525 | *** | -0.088 |  | -0.098 |  | -0.095 |  |
|  | (0.064) |  | (0.064) |  | (0.063) |  | (0.061) |  | (0.061) |  | (0.060) |  |
| Slovakia | -0.635 | *** | -0.641 | *** | -0.649 | *** | -0.247 | *** | -0.254 | *** | -0.242 | *** |
|  | (0.058) |  | (0.058) |  | (0.058) |  | (0.065) |  | (0.065) |  | (0.065) |  |
| Slovenia | -0.139 | ** | -0.149 | ** | -0.148 | ** | -0.096 | * | -0.099 | * | -0.105 | * |
|  | (0.052) |  | (0.051) |  | (0.051) |  | (0.048) |  | (0.047) |  | (0.047) |  |
| Spain | 0.008 |  | 0.012 |  | 0.009 |  | 0.332 | *** | 0.326 | *** | 0.331 | *** |
|  | (0.053) |  | (0.052) |  | (0.051) |  | (0.050) |  | (0.050) |  | (0.049) |  |
| Sweden | -0.237 | *** | -0.237 | *** | -0.234 | *** | -0.120 | * | -0.123 | * | -0.108 | * |
|  | (0.052) |  | (0.053) |  | (0.051) |  | (0.051) |  | (0.051) |  | (0.049) |  |
| Switzerland | -0.226 | *** | -0.233 | *** | -0.218 | *** | -0.201 | *** | -0.217 | *** | -0.233 | *** |
|  | (0.055) |  | (0.055) |  | (0.053) |  | (0.054) |  | (0.054) |  | (0.052) |  |
| Wave (ref: Wave 2) |  |  |  |  |  |  |  |  |  |  |  |  |
| Wave 4 | 0.073 | *** | 0.071 | *** | 0.066 | *** | 0.065 | *** | 0.065 | *** | 0.060 | *** |
|  | (0.015) |  | (0.015) |  | (0.015) |  | (0.015) |  | (0.015) |  | (0.015) |  |
| Wave 5 | 0.199 | *** | 0.202 | *** | 0.205 | *** | 0.214 | *** | 0.217 | *** | 0.220 | *** |
|  | (0.016) |  | (0.016) |  | (0.015) |  | (0.015) |  | (0.015) |  | (0.015) |  |
| Wave 6 | 0.324 | *** | 0.328 | *** | 0.337 | *** | 0.380 | *** | 0.380 | *** | 0.392 | *** |
|  | (0.017) |  | (0.017) |  | (0.016) |  | (0.016) |  | (0.016) |  | (0.016) |  |
| Wave 7 | 0.461 | *** | 0.463 | *** | 0.476 | *** | 0.575 | *** | 0.577 | *** | 0.577 | *** |
|  | (0.023) |  | (0.023) |  | (0.022) |  | (0.022) |  | (0.022) |  | (0.021) |  |
| Wave 8 | 0.491 | *** | 0.498 | *** | 0.512 | *** | 0.518 | *** | 0.522 | *** | 0.535 | *** |
|  | (0.019) |  | (0.019) |  | (0.018) |  | (0.018) |  | (0.018) |  | (0.018) |  |
| Wave 9 | 0.547 | *** | 0.552 | *** | 0.566 | *** | 0.545 | *** | 0.549 | *** | 0.560 | *** |
|  | (0.019) |  | (0.019) |  | (0.019) |  | (0.018) |  | (0.018) |  | (0.018) |  |
| Number of persons | 46,102 |  | 46,644 |  | 48,419 |  | 57,331 |  | 58,136 |  | 60,675 |  |
| Number of observations | 103,813 |  | 104,865 |  | 109,072 |  | 139,413 |  | 141,136 |  | 147,862 |  |
| R-squared | 0.180 |  | 0.181 |  | 0.181 |  | 0.213 |  | 0.214 |  | 0.215 |  |
| Inverse probability weights | 1.634 |  | 1.628 |  | 1.641 |  | 1.523 |  | 1.519 |  | 1.531 |  |
|  | (0.002) |  | (0.002) |  | (0.002) |  | (0.001) |  | (0.001) |  | (0.001) |  |

* p < 0.05; ** p < 0.01; *** p < 0.001

**Table S8.** Predicted number of chronic diseases by migration background and living arrangements for men and women based on origin-stratified analysis

|  | Men | | Women | |
| --- | --- | --- | --- | --- |
|  | Native-born | Immigrant | Native-born | Immigrant |
| Low HDI origin |  |  |  |  |
| Living alone | 1.80 (1.76, 1.84) | 2.18 (1.61, 2.74) | 2.15 (2.12, 2.18) | 2.48 (2.09, 2.86) |
| Living as a couple | 1.68 (1.67, 1.70) | 1.67 (1.43, 1.92) | 1.97 (1.95, 1.99) | 2.06 (1.79, 2.32) |
| Living as a family | 1.70 (1.67, 1.74) | 1.56 (1.23, 1.90) | 2.05 (2.02, 2.08) | 2.54 (2.25, 2.83) |
| Medium HDI origin |  |  |  |  |
| Living alone | 1.80 (1.76, 1.84) | 1.97 (1.61, 2.33) | 2.15 (2.12, 2.18) | 2.48 (2.28, 2.69) |
| Living as a couple | 1.68 (1.66, 1.70) | 1.91 (1.77, 2.06) | 1.97 (1.95, 1.99) | 2.33 (2.16, 2.49) |
| Living as a family | 1.70 (1.67, 1.74) | 1.76 (1.57, 1.96) | 2.05 (2.02, 2.08) | 2.55 (2.31, 2.78) |
| High HDI origin |  |  |  |  |
| Living alone | 1.80 (1.76, 1.84) | 1.78 (1.62, 1.94) | 2.15 (2.12, 2.18) | 2.49 (2.37, 2.61) |
| Living as a couple | 1.69 (1.67, 1.71) | 1.91 (1.83, 2.00) | 1.98 (1.95, 2.00) | 2.32 (2.23, 2.41) |
| Living as a family | 1.70 (1.67, 1.74) | 1.92 (1.76, 2.07) | 2.05 (2.02, 2.08) | 2.30 (2.16, 2.44) |

**Table S9.** Lagged-variable estimation of the role of living arrangements in chronic disease accumulation by migration background for men and women

|  | Men | | | | | | Women | | | | | |
| --- | --- | --- | --- | --- | --- | --- | --- | --- | --- | --- | --- | --- |
|  | Model 1 | | Model 2 | | Model 3 | | Model 1 | | Model 2 | | Model 3 | |
| (Intercept) | 1.850 | *** | 1.859 | *** | 1.827 | *** | 1.666 | *** | 1.674 | *** | 1.733 | *** |
|  | (0.085) |  | (0.086) |  | (0.118) |  | (0.073) |  | (0.074) |  | (0.107) |  |
| Immigrant (ref: native-born) | -0.035 |  | -0.150 |  | -0.326 |  | 0.070 |  | -0.007 |  | 0.266 |  |
|  | (0.153) |  | (0.176) |  | (0.381) |  | (0.134) |  | (0.158) |  | (0.328) |  |
| Age | 0.004 |  | 0.004 |  | 0.013 |  | 0.028 | *** | 0.028 | *** | 0.023 | * |
|  | (0.007) |  | (0.007) |  | (0.014) |  | (0.006) |  | (0.006) |  | (0.011) |  |
| Age^2^ | 0.001 | *** | 0.001 | *** | 0.001 |  | 0.001 | *** | 0.001 | *** | 0.001 | ** |
|  | (0.000) |  | (0.000) |  | (0.000) |  | (0.000) |  | (0.000) |  | (0.000) |  |
| Immigrant × age | 0.045 | * | 0.042 | * | 0.064 |  | 0.056 | ** | 0.059 | ** | 0.026 |  |
|  | (0.021) |  | (0.021) |  | (0.051) |  | (0.019) |  | (0.019) |  | (0.040) |  |
| Immigrant × age^2^ | -0.002 | * | -0.002 | * | -0.002 |  | -0.002 | ** | -0.002 | ** | -0.001 |  |
|  | (0.001) |  | (0.001) |  | (0.001) |  | (0.001) |  | (0.001) |  | (0.001) |  |
| Living arrangements (ref: Alone) |  |  |  |  |  |  |  |  |  |  |  |  |
| Living as a couple | -0.116 | *** | -0.136 | *** | 0.060 |  | -0.166 | *** | -0.171 | *** | 0.035 |  |
|  | (0.030) |  | (0.031) |  | (0.122) |  | (0.024) |  | (0.025) |  | (0.109) |  |
| Living as a family | -0.144 | *** | -0.157 | *** | -0.233 | * | -0.144 | *** | -0.154 | *** | -0.392 | *** |
|  | (0.034) |  | (0.035) |  | (0.118) |  | (0.028) |  | (0.029) |  | (0.104) |  |
| Immigrant × couple |  |  | 0.217 | * | 0.409 |  |  |  | 0.043 |  | -0.386 |  |
|  |  |  | (0.110) |  | (0.441) |  |  |  | (0.087) |  | (0.395) |  |
| Immigrant × family |  |  | 0.138 |  | 0.353 |  |  |  | 0.093 |  | -0.177 |  |
|  |  |  | (0.123) |  | (0.437) |  |  |  | (0.102) |  | (0.372) |  |
| Native-born × age × couple |  |  |  |  | -0.029 |  |  |  |  |  | -0.024 |  |
|  |  |  |  |  | (0.016) |  |  |  |  |  | (0.013) |  |
| Native-born × age × family |  |  |  |  | 0.002 |  |  |  |  |  | 0.017 |  |
|  |  |  |  |  | (0.017) |  |  |  |  |  | (0.014) |  |
| Native-born × age^2^ × couple |  |  |  |  | 0.001 |  |  |  |  |  | 0.001 |  |
|  |  |  |  |  | (0.000) |  |  |  |  |  | (0.000) |  |
| Native-born × age^2^ × family |  |  |  |  | 0.000 |  |  |  |  |  | -0.000 |  |
|  |  |  |  |  | (0.001) |  |  |  |  |  | (0.000) |  |
| Immigrant × age × couple |  |  |  |  | -0.057 |  |  |  |  |  | 0.032 |  |
|  |  |  |  |  | (0.056) |  |  |  |  |  | (0.047) |  |
| Immigrant × age × family |  |  |  |  | -0.020 |  |  |  |  |  | 0.046 |  |
|  |  |  |  |  | (0.060) |  |  |  |  |  | (0.049) |  |
| Immigrant × age^2^ × couple |  |  |  |  | 0.002 |  |  |  |  |  | -0.001 |  |
|  |  |  |  |  | (0.002) |  |  |  |  |  | (0.001) |  |
| Immigrant × age^2^ × family |  |  |  |  | 0.001 |  |  |  |  |  | -0.001 |  |
|  |  |  |  |  | (0.002) |  |  |  |  |  | (0.001) |  |
| Education (ref: Low) |  |  |  |  |  |  |  |  |  |  |  |  |
| Medium | -0.223 | *** | -0.223 | *** | -0.222 | *** | -0.337 | *** | -0.337 | *** | -0.331 | *** |
|  | (0.031) |  | (0.031) |  | (0.031) |  | (0.027) |  | (0.027) |  | (0.027) |  |
| High | -0.438 | *** | -0.436 | *** | -0.434 | *** | -0.591 | *** | -0.591 | *** | -0.582 | *** |
|  | (0.033) |  | (0.033) |  | (0.033) |  | (0.031) |  | (0.030) |  | (0.031) |  |
| Household income (ref: Low) |  |  |  |  |  |  |  |  |  |  |  |  |
| Medium | -0.046 |  | -0.047 | * | -0.045 |  | -0.114 | *** | -0.114 | *** | -0.103 | *** |
|  | (0.024) |  | (0.024) |  | (0.024) |  | (0.020) |  | (0.020) |  | (0.020) |  |
| High | -0.169 | *** | -0.170 | *** | -0.169 | *** | -0.180 | *** | -0.181 | *** | -0.176 | *** |
|  | (0.022) |  | (0.022) |  | (0.022) |  | (0.019) |  | (0.019) |  | (0.019) |  |
| Working (ref: Not working) | -0.593 | *** | -0.594 | *** | -0.596 | *** | -0.564 | *** | -0.564 | *** | -0.574 | *** |
|  | (0.028) |  | (0.028) |  | (0.028) |  | (0.026) |  | (0.026) |  | (0.026) |  |
| Have child (ref: No child) | 0.039 |  | 0.042 |  | 0.044 |  | 0.114 | *** | 0.114 | *** | 0.118 | *** |
|  | (0.031) |  | (0.031) |  | (0.031) |  | (0.029) |  | (0.029) |  | (0.029) |  |
| Country (ref: Austria) |  |  |  |  |  |  |  |  |  |  |  |  |
| Belgium | 0.254 | *** | 0.251 | *** | 0.254 | *** | 0.629 | *** | 0.629 | *** | 0.635 | *** |
|  | (0.066) |  | (0.066) |  | (0.066) |  | (0.061) |  | (0.061) |  | (0.061) |  |
| Bulgaria | -0.272 | ** | -0.273 | ** | -0.275 | ** | -0.001 |  | -0.001 |  | -0.016 |  |
|  | (0.100) |  | (0.100) |  | (0.100) |  | (0.085) |  | (0.085) |  | (0.085) |  |
| Croatia | -0.153 | * | -0.156 | * | -0.156 | * | 0.194 | ** | 0.193 | ** | 0.190 | ** |
|  | (0.075) |  | (0.075) |  | (0.075) |  | (0.072) |  | (0.072) |  | (0.072) |  |
| Cyprus | -0.392 | ** | -0.392 | ** | -0.390 | ** | -0.007 |  | -0.006 |  | 0.009 |  |
|  | (0.134) |  | (0.134) |  | (0.134) |  | (0.120) |  | (0.120) |  | (0.120) |  |
| Czech Republic | 0.350 | *** | 0.349 | *** | 0.349 | *** | 0.370 | *** | 0.370 | *** | 0.369 | *** |
|  | (0.073) |  | (0.073) |  | (0.073) |  | (0.060) |  | (0.060) |  | (0.060) |  |
| Denmark | 0.015 |  | 0.013 |  | 0.016 |  | 0.036 |  | 0.035 |  | 0.036 |  |
|  | (0.067) |  | (0.067) |  | (0.067) |  | (0.061) |  | (0.061) |  | (0.060) |  |
| Estonia | 0.280 | *** | 0.277 | *** | 0.277 | *** | 0.467 | *** | 0.468 | *** | 0.463 | *** |
|  | (0.069) |  | (0.069) |  | (0.069) |  | (0.058) |  | (0.058) |  | (0.058) |  |
| Finland | -0.253 | ** | -0.255 | ** | -0.255 | ** | -0.214 | ** | -0.215 | ** | -0.213 | * |
|  | (0.088) |  | (0.088) |  | (0.088) |  | (0.083) |  | (0.083) |  | (0.083) |  |
| France | -0.013 |  | -0.016 |  | -0.013 |  | 0.300 | *** | 0.300 | *** | 0.302 | *** |
|  | (0.068) |  | (0.068) |  | (0.068) |  | (0.061) |  | (0.061) |  | (0.061) |  |
| Germany | 0.165 | * | 0.164 | * | 0.164 | * | 0.168 | ** | 0.169 | ** | 0.170 | ** |
|  | (0.065) |  | (0.065) |  | (0.065) |  | (0.060) |  | (0.060) |  | (0.060) |  |
| Greece | -0.454 | *** | -0.456 | *** | -0.461 | *** | -0.172 | ** | -0.172 | ** | -0.175 | ** |
|  | (0.068) |  | (0.068) |  | (0.068) |  | (0.064) |  | (0.064) |  | (0.064) |  |
| Hungary | 0.425 | *** | 0.424 | *** | 0.422 | *** | 0.630 | *** | 0.630 | *** | 0.623 | *** |
|  | (0.108) |  | (0.108) |  | (0.108) |  | (0.092) |  | (0.092) |  | (0.092) |  |
| Italy | -0.035 |  | -0.036 |  | -0.036 |  | 0.218 | *** | 0.220 | *** | 0.226 | *** |
|  | (0.071) |  | (0.071) |  | (0.071) |  | (0.064) |  | (0.064) |  | (0.064) |  |
| Latvia | -0.296 | *** | -0.301 | *** | -0.302 | *** | 0.005 |  | 0.005 |  | -0.003 |  |
|  | (0.086) |  | (0.086) |  | (0.086) |  | (0.081) |  | (0.081) |  | (0.081) |  |
| Lithuania | 0.051 |  | 0.048 |  | 0.046 |  | 0.407 | *** | 0.406 | *** | 0.394 | *** |
|  | (0.097) |  | (0.097) |  | (0.097) |  | (0.077) |  | (0.077) |  | (0.077) |  |
| Luxembourg | 0.033 |  | 0.026 |  | 0.030 |  | 0.154 |  | 0.152 |  | 0.165 |  |
|  | (0.091) |  | (0.091) |  | (0.091) |  | (0.088) |  | (0.088) |  | (0.087) |  |
| Malta | -0.525 | *** | -0.526 | *** | -0.529 | *** | -0.529 | *** | -0.527 | *** | -0.527 | *** |
|  | (0.100) |  | (0.100) |  | (0.100) |  | (0.091) |  | (0.091) |  | (0.092) |  |
| Netherlands | -0.502 | *** | -0.504 | *** | -0.498 | *** | -0.385 | *** | -0.385 | *** | -0.373 | *** |
|  | (0.070) |  | (0.070) |  | (0.070) |  | (0.065) |  | (0.065) |  | (0.065) |  |
| Poland | 0.233 | ** | 0.231 | ** | 0.230 | ** | 0.493 | *** | 0.494 | *** | 0.484 | *** |
|  | (0.077) |  | (0.077) |  | (0.077) |  | (0.069) |  | (0.069) |  | (0.069) |  |
| Portugal | 0.268 |  | 0.268 |  | 0.264 |  | 0.944 | *** | 0.945 | *** | 0.948 | *** |
|  | (0.150) |  | (0.150) |  | (0.150) |  | (0.111) |  | (0.111) |  | (0.110) |  |
| Romania | -0.533 | *** | -0.534 | *** | -0.538 | *** | -0.004 |  | -0.003 |  | -0.020 |  |
|  | (0.086) |  | (0.086) |  | (0.087) |  | (0.082) |  | (0.082) |  | (0.082) |  |
| Slovakia | -0.609 | *** | -0.609 | *** | -0.607 | *** | -0.051 |  | -0.050 |  | -0.052 |  |
|  | (0.082) |  | (0.082) |  | (0.082) |  | (0.089) |  | (0.089) |  | (0.089) |  |
| Slovenia | -0.152 | * | -0.157 | * | -0.157 | * | -0.101 |  | -0.101 |  | -0.102 |  |
|  | (0.069) |  | (0.069) |  | (0.069) |  | (0.061) |  | (0.061) |  | (0.061) |  |
| Spain | -0.017 |  | -0.019 |  | -0.020 |  | 0.337 | *** | 0.338 | *** | 0.336 | *** |
|  | (0.071) |  | (0.071) |  | (0.071) |  | (0.066) |  | (0.066) |  | (0.066) |  |
| Sweden | -0.301 | *** | -0.303 | *** | -0.299 | *** | -0.180 | ** | -0.180 | ** | -0.168 | ** |
|  | (0.069) |  | (0.070) |  | (0.070) |  | (0.065) |  | (0.065) |  | (0.065) |  |
| Switzerland | -0.290 | *** | -0.290 | *** | -0.288 | *** | -0.293 | *** | -0.293 | *** | -0.285 | *** |
|  | (0.070) |  | (0.070) |  | (0.070) |  | (0.066) |  | (0.066) |  | (0.066) |  |
| Wave (ref: Wave 5) |  |  |  |  |  |  |  |  |  |  |  |  |
| Wave 6 | 0.080 | *** | 0.081 | *** | 0.081 | *** | 0.109 | *** | 0.109 | *** | 0.109 | *** |
|  | (0.016) |  | (0.016) |  | (0.016) |  | (0.012) |  | (0.012) |  | (0.012) |  |
| Wave 7 | 0.168 | *** | 0.170 | *** | 0.171 | *** | 0.241 | *** | 0.241 | *** | 0.247 | *** |
|  | (0.027) |  | (0.027) |  | (0.027) |  | (0.023) |  | (0.023) |  | (0.023) |  |
| Wave 8 | 0.247 | *** | 0.248 | *** | 0.247 | *** | 0.263 | *** | 0.263 | *** | 0.264 | *** |
|  | (0.023) |  | (0.023) |  | (0.023) |  | (0.019) |  | (0.019) |  | (0.019) |  |
| Wave 9 | 0.341 | *** | 0.341 | *** | 0.341 | *** | 0.301 | *** | 0.301 | *** | 0.304 | *** |
|  | (0.023) |  | (0.023) |  | (0.023) |  | (0.019) |  | (0.019) |  | (0.019) |  |
| Number of persons | 24,181 |  | 24,181 |  | 24,181 |  | 33,065 |  | 33,065 |  | 33,065 |  |
| Number of observations | 46,169 |  | 46,169 |  | 46,169 |  | 69,374 |  | 69,374 |  | 69,374 |  |
| R-squared | 0.152 |  | 0.152 |  | 0.152 |  | 0.190 |  | 0.190 |  | 0.191 |  |
| Inverse probability weights | 1.348 |  | 1.348 |  | 1.348 |  | 1.296 |  | 1.296 |  | 1.296 |  |
|  | (0.001) |  | (0.001) |  | (0.001) |  | (0.001) |  | (0.001) |  | (0.001) |  |

* p < 0.05; ** p < 0.01; *** p < 0.001

**Table S10.** Robustness checks for the impact of years since migration

|  | Men | | Women | |
| --- | --- | --- | --- | --- |
| (Intercept) | 1.251 | *** | 1.174 | *** |
|  | (0.068) |  | (0.062) |  |
| Immigrant (ref: native-born) | -0.214 |  | 0.180 |  |
|  | (0.194) |  | (0.176) |  |
| Age | 0.032 | *** | 0.035 | *** |
|  | (0.008) |  | (0.006) |  |
| Age^2^ | 0.000 |  | 0.001 | *** |
|  | (0.000) |  | (0.000) |  |
| Immigrant × age | 0.046 |  | 0.032 |  |
|  | (0.029) |  | (0.023) |  |
| Immigrant × age^2^ | -0.001 |  | -0.001 |  |
|  | (0.001) |  | (0.001) |  |
| Living arrangements (ref: Alone) |  |  |  |  |
| Living as a couple | 0.063 |  | -0.016 |  |
|  | (0.060) |  | (0.054) |  |
| Living as a family | -0.127 | * | -0.287 | *** |
|  | (0.057) |  | (0.052) |  |
| Immigrant × couple | 0.235 |  | -0.141 |  |
|  | (0.226) |  | (0.207) |  |
| Immigrant × family | 0.245 |  | -0.103 |  |
|  | (0.215) |  | (0.191) |  |
| Native-born × age × couple | -0.028 | ** | -0.017 | * |
|  | (0.009) |  | (0.007) |  |
| Native-born × age × family | -0.001 |  | 0.010 |  |
|  | (0.009) |  | (0.008) |  |
| Native-born × age^2^ × couple | 0.001 | ** | 0.000 |  |
|  | (0.000) |  | (0.000) |  |
| Native-born × age^2^ × family | 0.000 |  | 0.000 |  |
|  | (0.000) |  | (0.000) |  |
| Immigrant × age × couple | -0.040 |  | -0.002 |  |
|  | (0.032) |  | (0.027) |  |
| Immigrant × age × family | -0.025 |  | 0.030 |  |
|  | (0.033) |  | (0.028) |  |
| Immigrant × age^2^ × couple | 0.001 |  | 0.000 |  |
|  | (0.001) |  | (0.001) |  |
| Immigrant × age^2^ × family | 0.001 |  | -0.001 |  |
|  | (0.001) |  | (0.001) |  |
| Education (ref: Low) |  |  |  |  |
| Medium | -0.189 | *** | -0.282 | *** |
|  | (0.021) |  | (0.020) |  |
| High | -0.353 | *** | -0.500 | *** |
|  | (0.023) |  | (0.022) |  |
| Household income (ref: Low) |  |  |  |  |
| Medium | -0.023 |  | -0.060 | *** |
|  | (0.015) |  | (0.014) |  |
| High | -0.128 | *** | -0.146 | *** |
|  | (0.015) |  | (0.014) |  |
| Working (ref: Not working) | -0.573 | *** | -0.524 | *** |
|  | (0.018) |  | (0.017) |  |
| Have child (ref: No child) | 0.047 | * | 0.127 | *** |
|  | (0.023) |  | (0.021) |  |
| Country (ref: Austria) |  |  |  |  |
| Belgium | 0.269 | *** | 0.630 | *** |
|  | (0.047) |  | (0.046) |  |
| Bulgaria | -0.342 | *** | -0.101 |  |
|  | (0.067) |  | (0.060) |  |
| Croatia | -0.282 | *** | -0.049 |  |
|  | (0.050) |  | (0.049) |  |
| Cyprus | -0.462 | *** | -0.246 | *** |
|  | (0.079) |  | (0.074) |  |
| Czech Republic | 0.284 | *** | 0.352 | *** |
|  | (0.051) |  | (0.045) |  |
| Denmark | 0.078 |  | 0.131 | ** |
|  | (0.050) |  | (0.047) |  |
| Estonia | 0.306 | *** | 0.478 | *** |
|  | (0.050) |  | (0.045) |  |
| Finland | -0.246 | *** | -0.236 | *** |
|  | (0.059) |  | (0.056) |  |
| France | 0.041 |  | 0.342 | *** |
|  | (0.048) |  | (0.046) |  |
| Germany | 0.175 | *** | 0.159 | *** |
|  | (0.047) |  | (0.044) |  |
| Greece | -0.426 | *** | -0.178 | *** |
|  | (0.047) |  | (0.046) |  |
| Hungary | 0.294 | *** | 0.560 | *** |
|  | (0.058) |  | (0.056) |  |
| Ireland | -0.087 |  | -0.154 | * |
|  | (0.078) |  | (0.074) |  |
| Italy | -0.009 |  | 0.225 | *** |
|  | (0.050) |  | (0.047) |  |
| Latvia | -0.446 | *** | -0.153 | ** |
|  | (0.058) |  | (0.055) |  |
| Lithuania | -0.010 |  | 0.357 | *** |
|  | (0.073) |  | (0.060) |  |
| Luxembourg | 0.043 |  | 0.145 | * |
|  | (0.069) |  | (0.069) |  |
| Malta | -0.496 | *** | -0.519 | *** |
|  | (0.077) |  | (0.067) |  |
| Netherlands | -0.338 | *** | -0.265 | *** |
|  | (0.049) |  | (0.047) |  |
| Poland | 0.144 | ** | 0.367 | *** |
|  | (0.050) |  | (0.047) |  |
| Portugal | 0.137 | * | 0.722 | *** |
|  | (0.068) |  | (0.065) |  |
| Romania | -0.536 | *** | -0.097 |  |
|  | (0.063) |  | (0.060) |  |
| Slovakia | -0.659 | *** | -0.243 | *** |
|  | (0.058) |  | (0.065) |  |
| Slovenia | -0.159 | ** | -0.097 | * |
|  | (0.050) |  | (0.046) |  |
| Spain | -0.008 |  | 0.322 | *** |
|  | (0.051) |  | (0.049) |  |
| Sweden | -0.235 | *** | -0.103 | * |
|  | (0.051) |  | (0.049) |  |
| Switzerland | -0.217 | *** | -0.225 | *** |
|  | (0.053) |  | (0.051) |  |
| Wave (ref: Wave 2) |  |  |  |  |
| Wave 4 | 0.062 | *** | 0.063 | *** |
|  | (0.015) |  | (0.014) |  |
| Wave 5 | 0.201 | *** | 0.222 | *** |
|  | (0.015) |  | (0.015) |  |
| Wave 6 | 0.336 | *** | 0.392 | *** |
|  | (0.016) |  | (0.016) |  |
| Wave 7 | 0.475 | *** | 0.582 | *** |
|  | (0.022) |  | (0.021) |  |
| Wave 8 | 0.511 | *** | 0.541 | *** |
|  | (0.018) |  | (0.018) |  |
| Wave 9 | 0.565 | *** | 0.566 | *** |
|  | (0.018) |  | (0.018) |  |
| Number of persons | 49,791 |  | 62,234 |  |
| Number of observations | 111,821 |  | 151,312 |  |
| R-squared | 0.181 |  | 0.215 |  |
| Inverse probability weights | 1.646 |  | 1.531 |  |
|  | (0.002) |  | (0.001) |  |

* p < 0.05; ** p < 0.01; *** p < 0.001

Table S11. Robustness checks on country of origin groupings using gross national income (GNI) per capita

|  | Men | | | | | | Women | | | | | |
| --- | --- | --- | --- | --- | --- | --- | --- | --- | --- | --- | --- | --- |
|  | Low | | Medium | | High | | Low | | Medium | | High | |
| (Intercept) | 1.224 | *** | 1.234 | *** | 1.237 | *** | 1.162 | *** | 1.179 | *** | 1.172 | *** |
|  | (0.069) |  | (0.068) |  | (0.068) |  | (0.063) |  | (0.063) |  | (0.063) |  |
| Immigrant (ref: Native-born) | 0.326 |  | -0.486 | * | -0.323 |  | -0.627 |  | 0.238 |  | 0.152 |  |
|  | (0.564) |  | (0.244) |  | (0.224) |  | (0.441) |  | (0.257) |  | (0.246) |  |
| Age | 0.033 | *** | 0.032 | *** | 0.032 | *** | 0.036 | *** | 0.036 | *** | 0.036 | *** |
|  | (0.008) |  | (0.008) |  | (0.008) |  | (0.006) |  | (0.006) |  | (0.006) |  |
| Age^2^ | 0.000 |  | 0.000 |  | 0.000 |  | 0.001 | ** | 0.001 | ** | 0.001 | ** |
|  | (0.000) |  | (0.000) |  | (0.000) |  | (0.000) |  | (0.000) |  | (0.000) |  |
| Immigrant × age | 0.026 |  | 0.117 | ** | 0.025 |  | 0.143 | * | 0.047 |  | 0.010 |  |
|  | (0.081) |  | (0.042) |  | (0.037) |  | (0.060) |  | (0.032) |  | (0.032) |  |
| Immigrant × age^2^ | -0.002 |  | -0.004 | ** | -0.000 |  | -0.004 | * | -0.001 |  | -0.001 |  |
|  | (0.003) |  | (0.001) |  | (0.001) |  | (0.002) |  | (0.001) |  | (0.001) |  |
| Living arrangements (ref: Alone) |  |  |  |  |  |  |  |  |  |  |  |  |
| Living as a couple | 0.055 |  | 0.058 |  | 0.058 |  | -0.019 |  | -0.018 |  | -0.019 |  |
|  | (0.060) |  | (0.060) |  | (0.060) |  | (0.054) |  | (0.054) |  | (0.054) |  |
| Living as a family | -0.134 | * | -0.132 | * | -0.135 | * | -0.294 | *** | -0.292 | *** | -0.293 | *** |
|  | (0.057) |  | (0.057) |  | (0.057) |  | (0.052) |  | (0.052) |  | (0.052) |  |
| Immigrant × couple | -0.284 |  | 0.489 |  | 0.294 |  | 0.137 |  | -0.346 |  | -0.015 |  |
|  | (0.628) |  | (0.293) |  | (0.282) |  | (0.520) |  | (0.311) |  | (0.285) |  |
| Immigrant × family | -0.294 |  | 0.550 | * | 0.208 |  | 0.709 |  | -0.036 |  | -0.288 |  |
|  | (0.605) |  | (0.268) |  | (0.272) |  | (0.460) |  | (0.281) |  | (0.271) |  |
| Native-born × age × couple | -0.027 | ** | -0.028 | ** | -0.028 | ** | -0.017 | * | -0.017 | * | -0.017 | * |
|  | (0.009) |  | (0.009) |  | (0.009) |  | (0.007) |  | (0.007) |  | (0.007) |  |
| Native-born × age × family | -0.000 |  | -0.000 |  | -0.001 |  | 0.011 |  | 0.010 |  | 0.010 |  |
|  | (0.009) |  | (0.009) |  | (0.009) |  | (0.008) |  | (0.008) |  | (0.008) |  |
| Native-born × age^2^ × couple | 0.001 | ** | 0.001 | ** | 0.001 | ** | 0.000 |  | 0.000 |  | 0.000 |  |
|  | (0.000) |  | (0.000) |  | (0.000) |  | (0.000) |  | (0.000) |  | (0.000) |  |
| Native-born × age^2^ × family | 0.000 |  | 0.000 |  | 0.000 |  | 0.000 |  | 0.000 |  | 0.000 |  |
|  | (0.000) |  | (0.000) |  | (0.000) |  | (0.000) |  | (0.000) |  | (0.000) |  |
| mmigrant × age × couple | -0.048 |  | -0.105 | * | -0.013 |  | -0.064 |  | 0.000 |  | 0.003 |  |
|  | (0.092) |  | (0.047) |  | (0.042) |  | (0.075) |  | (0.041) |  | (0.038) |  |
| Immigrant × age × family | -0.016 |  | -0.104 | * | 0.022 |  | -0.117 |  | 0.020 |  | 0.063 |  |
|  | (0.095) |  | (0.049) |  | (0.046) |  | (0.067) |  | (0.041) |  | (0.041) |  |
| Immigrant × age^2^ × couple | 0.003 |  | 0.004 | * | -0.000 |  | 0.002 |  | 0.000 |  | -0.000 |  |
|  | (0.003) |  | (0.002) |  | (0.001) |  | (0.002) |  | (0.001) |  | (0.001) |  |
| Immigrant × age^2^ × family | 0.001 |  | 0.003 | * | -0.001 |  | 0.005 | * | -0.001 |  | -0.002 |  |
|  | (0.003) |  | (0.002) |  | (0.002) |  | (0.002) |  | (0.001) |  | (0.001) |  |
| Education (ref: Low) |  |  |  |  |  |  |  |  |  |  |  |  |
| Medium | -0.188 | *** | -0.193 | *** | -0.193 | *** | -0.278 | *** | -0.283 | *** | -0.285 | *** |
|  | (0.022) |  | (0.022) |  | (0.022) |  | (0.020) |  | (0.020) |  | (0.020) |  |
| High | -0.345 | *** | -0.345 | *** | -0.358 | *** | -0.490 | *** | -0.495 | *** | -0.500 | *** |
|  | (0.024) |  | (0.024) |  | (0.024) |  | (0.023) |  | (0.023) |  | (0.023) |  |
| Household income (ref: Low) |  |  |  |  |  |  |  |  |  |  |  |  |
| Medium | -0.023 |  | -0.022 |  | -0.021 |  | -0.049 | *** | -0.052 | *** | -0.058 | *** |
|  | (0.016) |  | (0.016) |  | (0.016) |  | (0.014) |  | (0.014) |  | (0.014) |  |
| High | -0.121 | *** | -0.122 | *** | -0.127 | *** | -0.137 | *** | -0.141 | *** | -0.142 | *** |
|  | (0.015) |  | (0.015) |  | (0.015) |  | (0.014) |  | (0.014) |  | (0.014) |  |
| Working (ref: Not working) | -0.555 | *** | -0.563 | *** | -0.563 | *** | -0.511 | *** | -0.511 | *** | -0.511 | *** |
|  | (0.018) |  | (0.018) |  | (0.018) |  | (0.017) |  | (0.017) |  | (0.017) |  |
| Have child (ref: No child) | 0.043 |  | 0.044 |  | 0.052 | * | 0.131 | *** | 0.132 | *** | 0.128 | *** |
|  | (0.023) |  | (0.023) |  | (0.023) |  | (0.022) |  | (0.022) |  | (0.022) |  |
| Country (ref: Austria) |  |  |  |  |  |  |  |  |  |  |  |  |
| Belgium | 0.297 | *** | 0.287 | *** | 0.279 | *** | 0.643 | *** | 0.627 | *** | 0.639 | *** |
|  | (0.049) |  | (0.049) |  | (0.048) |  | (0.047) |  | (0.047) |  | (0.046) |  |
| Bulgaria | -0.318 | *** | -0.328 | *** | -0.330 | *** | -0.098 |  | -0.112 |  | -0.105 |  |
|  | (0.068) |  | (0.068) |  | (0.068) |  | (0.061) |  | (0.061) |  | (0.060) |  |
| Croatia | -0.221 | *** | -0.271 | *** | -0.237 | *** | 0.008 |  | -0.077 |  | -0.002 |  |
|  | (0.052) |  | (0.050) |  | (0.052) |  | (0.052) |  | (0.050) |  | (0.051) |  |
| Cyprus | -0.453 | *** | -0.462 | *** | -0.457 | *** | -0.234 | ** | -0.251 | *** | -0.248 | *** |
|  | (0.080) |  | (0.079) |  | (0.079) |  | (0.076) |  | (0.076) |  | (0.075) |  |
| Czech Republic | 0.300 | *** | 0.291 | *** | 0.289 | *** | 0.357 | *** | 0.343 | *** | 0.352 | *** |
|  | (0.052) |  | (0.051) |  | (0.051) |  | (0.046) |  | (0.046) |  | (0.045) |  |
| Denmark | 0.090 |  | 0.091 |  | 0.081 |  | 0.122 | * | 0.110 | * | 0.126 | ** |
|  | (0.051) |  | (0.051) |  | (0.051) |  | (0.048) |  | (0.048) |  | (0.048) |  |
| Estonia | 0.314 | *** | 0.298 | *** | 0.284 | *** | 0.408 | *** | 0.416 | *** | 0.405 | *** |
|  | (0.053) |  | (0.051) |  | (0.053) |  | (0.047) |  | (0.046) |  | (0.047) |  |
| Finland | -0.226 | *** | -0.237 | *** | -0.232 | *** | -0.237 | *** | -0.251 | *** | -0.241 | *** |
|  | (0.060) |  | (0.060) |  | (0.060) |  | (0.057) |  | (0.056) |  | (0.056) |  |
| France | 0.051 |  | 0.044 |  | 0.057 |  | 0.340 | *** | 0.326 | *** | 0.340 | *** |
|  | (0.050) |  | (0.049) |  | (0.049) |  | (0.047) |  | (0.047) |  | (0.046) |  |
| Germany | 0.211 | *** | 0.205 | *** | 0.194 | *** | 0.158 | *** | 0.148 | *** | 0.161 | *** |
|  | (0.049) |  | (0.048) |  | (0.048) |  | (0.045) |  | (0.045) |  | (0.045) |  |
| Greece | -0.412 | *** | -0.419 | *** | -0.424 | *** | -0.178 | *** | -0.191 | *** | -0.184 | *** |
|  | (0.048) |  | (0.047) |  | (0.047) |  | (0.047) |  | (0.047) |  | (0.046) |  |
| Hungary | 0.307 | *** | 0.305 | *** | 0.300 | *** | 0.561 | *** | 0.551 | *** | 0.557 | *** |
|  | (0.059) |  | (0.059) |  | (0.059) |  | (0.056) |  | (0.056) |  | (0.056) |  |
| Ireland | -0.094 |  | -0.090 |  | -0.094 |  | -0.195 | ** | -0.205 | ** | -0.161 | * |
|  | (0.080) |  | (0.080) |  | (0.077) |  | (0.075) |  | (0.075) |  | (0.074) |  |
| Italy | 0.002 |  | -0.002 |  | -0.003 |  | 0.231 | *** | 0.212 | *** | 0.226 | *** |
|  | (0.051) |  | (0.051) |  | (0.051) |  | (0.048) |  | (0.047) |  | (0.047) |  |
| Latvia | -0.397 | *** | -0.440 | *** | -0.431 | *** | -0.114 | * | -0.190 | *** | -0.120 | * |
|  | (0.062) |  | (0.060) |  | (0.062) |  | (0.057) |  | (0.056) |  | (0.057) |  |
| Lithuania | -0.000 |  | -0.010 |  | -0.014 |  | 0.358 | *** | 0.333 | *** | 0.347 | *** |
|  | (0.075) |  | (0.073) |  | (0.074) |  | (0.062) |  | (0.061) |  | (0.061) |  |
| Luxembourg | 0.118 |  | 0.113 |  | 0.073 |  | 0.229 | ** | 0.196 | * | 0.161 | * |
|  | (0.083) |  | (0.083) |  | (0.072) |  | (0.080) |  | (0.080) |  | (0.070) |  |
| Malta | -0.490 | *** | -0.503 | *** | -0.485 | *** | -0.508 | *** | -0.528 | *** | -0.516 | *** |
|  | (0.075) |  | (0.075) |  | (0.077) |  | (0.068) |  | (0.068) |  | (0.068) |  |
| Netherlands | -0.333 | *** | -0.341 | *** | -0.330 | *** | -0.274 | *** | -0.286 | *** | -0.271 | *** |
|  | (0.050) |  | (0.050) |  | (0.050) |  | (0.048) |  | (0.048) |  | (0.047) |  |
| Poland | 0.162 | ** | 0.154 | ** | 0.155 | ** | 0.369 | *** | 0.355 | *** | 0.369 | *** |
|  | (0.051) |  | (0.051) |  | (0.051) |  | (0.048) |  | (0.048) |  | (0.047) |  |
| Portugal | 0.164 | * | 0.153 | * | 0.148 | * | 0.730 | *** | 0.733 | *** | 0.739 | *** |
|  | (0.069) |  | (0.069) |  | (0.069) |  | (0.065) |  | (0.066) |  | (0.066) |  |
| Romania | -0.509 | *** | -0.519 | *** | -0.520 | *** | -0.087 |  | -0.106 |  | -0.094 |  |
|  | (0.064) |  | (0.063) |  | (0.063) |  | (0.061) |  | (0.061) |  | (0.060) |  |
| Slovakia | -0.635 | *** | -0.644 | *** | -0.645 | *** | -0.247 | *** | -0.264 | *** | -0.240 | *** |
|  | (0.058) |  | (0.058) |  | (0.058) |  | (0.065) |  | (0.065) |  | (0.065) |  |
| Slovenia | -0.138 | ** | -0.155 | ** | -0.144 | ** | -0.096 | * | -0.106 | * | -0.103 | * |
|  | (0.052) |  | (0.051) |  | (0.051) |  | (0.048) |  | (0.047) |  | (0.047) |  |
| Spain | 0.008 |  | 0.006 |  | 0.012 |  | 0.333 | *** | 0.318 | *** | 0.336 | *** |
|  | (0.053) |  | (0.052) |  | (0.052) |  | (0.050) |  | (0.050) |  | (0.049) |  |
| Sweden | -0.239 | *** | -0.233 | *** | -0.234 | *** | -0.121 | * | -0.127 | * | -0.099 | * |
|  | (0.052) |  | (0.052) |  | (0.052) |  | (0.051) |  | (0.051) |  | (0.049) |  |
| Switzerland | -0.228 | *** | -0.215 | *** | -0.228 | *** | -0.204 | *** | -0.226 | *** | -0.212 | *** |
|  | (0.055) |  | (0.056) |  | (0.053) |  | (0.054) |  | (0.053) |  | (0.052) |  |
| Wave (ref: Wave 2) |  |  |  |  |  |  |  |  |  |  |  |  |
| Wave 4 | 0.069 | *** | 0.066 | *** | 0.072 | *** | 0.065 | *** | 0.055 | *** | 0.069 | *** |
|  | (0.015) |  | (0.015) |  | (0.015) |  | (0.015) |  | (0.015) |  | (0.015) |  |
| Wave 5 | 0.197 | *** | 0.205 | *** | 0.203 | *** | 0.215 | *** | 0.217 | *** | 0.219 | *** |
|  | (0.016) |  | (0.016) |  | (0.015) |  | (0.015) |  | (0.015) |  | (0.015) |  |
| Wave 6 | 0.324 | *** | 0.332 | *** | 0.333 | *** | 0.380 | *** | 0.385 | *** | 0.386 | *** |
|  | (0.017) |  | (0.017) |  | (0.016) |  | (0.016) |  | (0.016) |  | (0.016) |  |
| Wave 7 | 0.458 | *** | 0.468 | *** | 0.472 | *** | 0.574 | *** | 0.578 | *** | 0.577 | *** |
|  | (0.023) |  | (0.023) |  | (0.022) |  | (0.022) |  | (0.022) |  | (0.021) |  |
| Wave 8 | 0.490 | *** | 0.508 | *** | 0.502 | *** | 0.516 | *** | 0.529 | *** | 0.529 | *** |
|  | (0.019) |  | (0.019) |  | (0.018) |  | (0.018) |  | (0.018) |  | (0.018) |  |
| Wave 9 | 0.546 | *** | 0.560 | *** | 0.558 | *** | 0.545 | *** | 0.552 | *** | 0.557 | *** |
|  | (0.019) |  | (0.019) |  | (0.019) |  | (0.018) |  | (0.018) |  | (0.018) |  |
| Number of persons | 46,233 |  | 47,360 |  | 47,569 |  | 57,498 |  | 59,334 |  | 59,308 |  |
| Number of observations | 104,112 |  | 106,307 |  | 107,332 |  | 139,831 |  | 144,048 |  | 144,526 |  |
| R-squared | 0.180 |  | 0.181 |  | 0.181 |  | 0.214 |  | 0.216 |  | 0.213 |  |
| Inverse probability weights | 1.633 |  | 1.632 |  | 1.638 |  | 1.523 |  | 1.521 |  | 1.529 |  |
|  | (0.002) |  | (0.002) |  | (0.002) |  | (0.001) |  | (0.001) |  | (0.001) |  |

* p < 0.05; ** p < 0.01; *** p < 0.001

Table S12. Robustness checks on living arrangements categorization by distinguishing living as a family with versus without spouse or partner

|  | Men | | | | | | Women | | | | | |
| --- | --- | --- | --- | --- | --- | --- | --- | --- | --- | --- | --- | --- |
|  | Model 1 | | Model 2 | | Model 3 | | Model 1 | | Model 2 | | Model 3 | |
| (Intercept) | 1.295 | *** | 1.301 | *** | 1.247 | *** | 1.169 | *** | 1.169 | *** | 1.167 | *** |
|  | (0.054) |  | (0.055) |  | (0.068) |  | (0.048) |  | (0.049) |  | (0.062) |  |
| Immigrant (ref: Native-born) | -0.074 |  | -0.145 |  | -0.218 |  | 0.041 |  | 0.043 |  | 0.097 |  |
|  | (0.069) |  | (0.097) |  | (0.186) |  | (0.065) |  | (0.088) |  | (0.171) |  |
| Age | 0.020 | *** | 0.020 | *** | 0.032 | *** | 0.035 | *** | 0.035 | *** | 0.035 | *** |
|  | (0.003) |  | (0.003) |  | (0.008) |  | (0.003) |  | (0.003) |  | (0.006) |  |
| Age^2^ | 0.001 | *** | 0.001 | *** | 0.000 |  | 0.001 | *** | 0.001 | *** | 0.001 | *** |
|  | (0.000) |  | (0.000) |  | (0.000) |  | (0.000) |  | (0.000) |  | (0.000) |  |
| Immigrant × age | 0.039 | *** | 0.035 | ** | 0.046 |  | 0.046 | *** | 0.046 | *** | 0.038 |  |
|  | (0.011) |  | (0.011) |  | (0.028) |  | (0.010) |  | (0.011) |  | (0.022) |  |
| Immigrant × age^2^ | -0.001 | *** | -0.001 | ** | -0.001 |  | -0.001 | *** | -0.001 | *** | -0.001 |  |
|  | (0.000) |  | (0.000) |  | (0.001) |  | (0.000) |  | (0.000) |  | (0.001) |  |
| Living arrangements (ref: Alone) |  |  |  |  |  |  |  |  |  |  |  |  |
| Living as a couple | -0.098 | *** | -0.112 | *** | 0.063 |  | -0.180 | *** | -0.179 | *** | -0.019 |  |
|  | (0.022) |  | (0.022) |  | (0.060) |  | (0.018) |  | (0.018) |  | (0.054) |  |
| Living as a family w/ spouse | -0.105 | *** | -0.110 | *** | -0.119 | * | -0.213 | *** | -0.213 | *** | -0.304 | *** |
|  | (0.025) |  | (0.026) |  | (0.058) |  | (0.022) |  | (0.023) |  | (0.054) |  |
| Living as a family w/o spouse | -0.042 |  | -0.061 |  | -0.204 | * | -0.006 |  | -0.006 |  | -0.193 | ** |
|  | (0.043) |  | (0.044) |  | (0.102) |  | (0.028) |  | (0.029) |  | (0.070) |  |
| Immigrant × couple |  |  | 0.157 |  | 0.197 |  |  |  | -0.004 |  | -0.110 |  |
|  |  |  | (0.082) |  | (0.216) |  |  |  | (0.064) |  | (0.200) |  |
| Immigrant × family w/ spouse |  |  | 0.060 |  | 0.236 |  |  |  | -0.003 |  | 0.055 |  |
|  |  |  | (0.092) |  | (0.207) |  |  |  | (0.081) |  | (0.196) |  |
| Immigrant × family w/o spouse |  |  | 0.232 |  | -0.059 |  |  |  | -0.002 |  | -0.254 |  |
|  |  |  | (0.178) |  | (0.390) |  |  |  | (0.098) |  | (0.236) |  |
| Native-born × age × couple |  |  |  |  | -0.028 | ** |  |  |  |  | -0.017 | * |
|  |  |  |  |  | (0.009) |  |  |  |  |  | (0.007) |  |
| Native-born × age × family w/ spouse |  |  |  |  | -0.002 |  |  |  |  |  | 0.005 |  |
|  |  |  |  |  | (0.009) |  |  |  |  |  | (0.008) |  |
| Native-born × age × family w/o spouse |  |  |  |  | 0.013 |  |  |  |  |  | 0.015 |  |
|  |  |  |  |  | (0.017) |  |  |  |  |  | (0.011) |  |
| Native-born × age^2^ × couple |  |  |  |  | 0.001 | ** |  |  |  |  | 0.000 |  |
|  |  |  |  |  | (0.000) |  |  |  |  |  | (0.000) |  |
| Native-born × age^2^ × family w/ spouse |  |  |  |  | 0.000 |  |  |  |  |  | 0.000 |  |
|  |  |  |  |  | (0.000) |  |  |  |  |  | (0.000) |  |
| Native-born × age^2^ × family w/o spouse |  |  |  |  | -0.000 |  |  |  |  |  | -0.000 |  |
|  |  |  |  |  | (0.001) |  |  |  |  |  | (0.000) |  |
| Immigrant × age × couple |  |  |  |  | -0.035 |  |  |  |  |  | -0.005 |  |
|  |  |  |  |  | (0.031) |  |  |  |  |  | (0.026) |  |
| Immigrant × age × family w/ spouse |  |  |  |  | -0.030 |  |  |  |  |  | -0.004 |  |
|  |  |  |  |  | (0.032) |  |  |  |  |  | (0.030) |  |
| Immigrant × age × family w/o spouse |  |  |  |  | 0.068 |  |  |  |  |  | 0.067 |  |
|  |  |  |  |  | (0.070) |  |  |  |  |  | (0.036) |  |
| Immigrant × age^2^ × couple |  |  |  |  | 0.001 |  |  |  |  |  | 0.000 |  |
|  |  |  |  |  | (0.001) |  |  |  |  |  | (0.001) |  |
| Immigrant × age^2^ × family w/ spouse |  |  |  |  | 0.001 |  |  |  |  |  | 0.000 |  |
|  |  |  |  |  | (0.001) |  |  |  |  |  | (0.001) |  |
| Immigrant × age^2^ × family w/o spouse |  |  |  |  | -0.002 |  |  |  |  |  | -0.002 |  |
|  |  |  |  |  | (0.002) |  |  |  |  |  | (0.001) |  |
| Education (ref: Low) |  |  |  |  |  |  |  |  |  |  |  |  |
| Medium | -0.189 | *** | -0.189 | *** | -0.188 | *** | -0.285 | *** | -0.285 | *** | -0.280 | *** |
|  | (0.021) |  | (0.021) |  | (0.021) |  | (0.020) |  | (0.020) |  | (0.020) |  |
| High | -0.355 | *** | -0.354 | *** | -0.352 | *** | -0.508 | *** | -0.508 | *** | -0.500 | *** |
|  | (0.023) |  | (0.023) |  | (0.023) |  | (0.022) |  | (0.022) |  | (0.022) |  |
| Household income (ref: Low) |  |  |  |  |  |  |  |  |  |  |  |  |
| Medium | -0.024 |  | -0.024 |  | -0.022 |  | -0.054 | *** | -0.054 | *** | -0.048 | *** |
|  | (0.015) |  | (0.015) |  | (0.015) |  | (0.014) |  | (0.014) |  | (0.014) |  |
| High | -0.126 | *** | -0.127 | *** | -0.125 | *** | -0.133 | *** | -0.133 | *** | -0.133 | *** |
|  | (0.015) |  | (0.015) |  | (0.015) |  | (0.014) |  | (0.014) |  | (0.014) |  |
| Working (ref: Not working) | -0.569 | *** | -0.569 | *** | -0.574 | *** | -0.518 | *** | -0.518 | *** | -0.526 | *** |
|  | (0.018) |  | (0.018) |  | (0.018) |  | (0.017) |  | (0.017) |  | (0.017) |  |
| Have child (ref: No child) | 0.045 | * | 0.047 | * | 0.049 | * | 0.125 | *** | 0.125 | *** | 0.129 | *** |
|  | (0.023) |  | (0.023) |  | (0.023) |  | (0.021) |  | (0.021) |  | (0.021) |  |
| Country (ref: Austria) |  |  |  |  |  |  |  |  |  |  |  |  |
| Belgium | 0.175 | *** | 0.175 | *** | 0.176 | *** | 0.158 | *** | 0.158 | *** | 0.160 | *** |
|  | (0.047) |  | (0.047) |  | (0.047) |  | (0.044) |  | (0.044) |  | (0.044) |  |
| Bulgaria | -0.237 | *** | -0.238 | *** | -0.234 | *** | -0.110 | * | -0.110 | * | -0.099 | * |
|  | (0.051) |  | (0.051) |  | (0.051) |  | (0.049) |  | (0.049) |  | (0.049) |  |
| Croatia | -0.342 | *** | -0.342 | *** | -0.337 | *** | -0.270 | *** | -0.271 | *** | -0.262 | *** |
|  | (0.049) |  | (0.049) |  | (0.049) |  | (0.047) |  | (0.047) |  | (0.047) |  |
| Cyprus | -0.010 |  | -0.011 |  | -0.010 |  | 0.319 | *** | 0.319 | *** | 0.319 | *** |
|  | (0.051) |  | (0.051) |  | (0.051) |  | (0.049) |  | (0.049) |  | (0.049) |  |
| Czech Republic | -0.006 |  | -0.007 |  | -0.006 |  | 0.227 | *** | 0.227 | *** | 0.232 | *** |
|  | (0.050) |  | (0.050) |  | (0.050) |  | (0.047) |  | (0.047) |  | (0.047) |  |
| Denmark | 0.040 |  | 0.039 |  | 0.041 |  | 0.336 | *** | 0.336 | *** | 0.341 | *** |
|  | (0.048) |  | (0.048) |  | (0.048) |  | (0.046) |  | (0.046) |  | (0.046) |  |
| Estonia | 0.078 |  | 0.077 |  | 0.080 |  | 0.132 | ** | 0.132 | ** | 0.133 | ** |
|  | (0.050) |  | (0.050) |  | (0.050) |  | (0.047) |  | (0.047) |  | (0.047) |  |
| Finland | -0.421 | *** | -0.423 | *** | -0.423 | *** | -0.179 | *** | -0.179 | *** | -0.177 | *** |
|  | (0.047) |  | (0.047) |  | (0.047) |  | (0.046) |  | (0.046) |  | (0.046) |  |
| France | -0.214 | *** | -0.213 | *** | -0.211 | *** | -0.226 | *** | -0.226 | *** | -0.219 | *** |
|  | (0.053) |  | (0.053) |  | (0.053) |  | (0.051) |  | (0.051) |  | (0.051) |  |
| Germany | 0.269 | *** | 0.268 | *** | 0.271 | *** | 0.620 | *** | 0.620 | *** | 0.627 | *** |
|  | (0.047) |  | (0.047) |  | (0.047) |  | (0.046) |  | (0.046) |  | (0.046) |  |
| Greece | 0.284 | *** | 0.283 | *** | 0.284 | *** | 0.347 | *** | 0.347 | *** | 0.349 | *** |
|  | (0.051) |  | (0.051) |  | (0.051) |  | (0.045) |  | (0.045) |  | (0.045) |  |
| Hungary | 0.146 | ** | 0.145 | ** | 0.145 | ** | 0.372 | *** | 0.372 | *** | 0.366 | *** |
|  | (0.050) |  | (0.050) |  | (0.050) |  | (0.047) |  | (0.047) |  | (0.047) |  |
| Ireland | -0.104 |  | -0.107 |  | -0.103 |  | -0.168 | * | -0.168 | * | -0.164 | * |
|  | (0.076) |  | (0.076) |  | (0.076) |  | (0.073) |  | (0.073) |  | (0.074) |  |
| Italy | 0.045 |  | 0.043 |  | 0.046 |  | 0.145 | * | 0.145 | * | 0.156 | * |
|  | (0.069) |  | (0.069) |  | (0.069) |  | (0.069) |  | (0.069) |  | (0.069) |  |
| Latvia | 0.296 | *** | 0.296 | *** | 0.296 | *** | 0.561 | *** | 0.561 | *** | 0.559 | *** |
|  | (0.058) |  | (0.058) |  | (0.058) |  | (0.056) |  | (0.056) |  | (0.056) |  |
| Lithuania | 0.142 | * | 0.141 | * | 0.141 | * | 0.727 | *** | 0.727 | *** | 0.728 | *** |
|  | (0.068) |  | (0.068) |  | (0.068) |  | (0.065) |  | (0.065) |  | (0.064) |  |
| Luxembourg | -0.157 | ** | -0.160 | ** | -0.159 | ** | -0.091 | * | -0.091 | * | -0.092 | * |
|  | (0.050) |  | (0.050) |  | (0.050) |  | (0.046) |  | (0.046) |  | (0.046) |  |
| Malta | 0.308 | *** | 0.306 | *** | 0.307 | *** | 0.480 | *** | 0.480 | *** | 0.480 | *** |
|  | (0.050) |  | (0.050) |  | (0.050) |  | (0.045) |  | (0.045) |  | (0.045) |  |
| Netherlands | -0.283 | *** | -0.284 | *** | -0.284 | *** | -0.042 |  | -0.042 |  | -0.043 |  |
|  | (0.050) |  | (0.050) |  | (0.050) |  | (0.049) |  | (0.049) |  | (0.049) |  |
| Poland | -0.006 |  | -0.007 |  | -0.009 |  | 0.364 | *** | 0.364 | *** | 0.358 | *** |
|  | (0.073) |  | (0.073) |  | (0.073) |  | (0.060) |  | (0.060) |  | (0.060) |  |
| Portugal | -0.340 | *** | -0.340 | *** | -0.341 | *** | -0.097 |  | -0.097 |  | -0.103 |  |
|  | (0.067) |  | (0.067) |  | (0.067) |  | (0.060) |  | (0.060) |  | (0.060) |  |
| Romania | -0.461 | *** | -0.461 | *** | -0.457 | *** | -0.251 | *** | -0.251 | *** | -0.238 | ** |
|  | (0.079) |  | (0.079) |  | (0.079) |  | (0.074) |  | (0.074) |  | (0.074) |  |
| Slovakia | -0.243 | *** | -0.244 | *** | -0.243 | *** | -0.239 | *** | -0.239 | *** | -0.233 | *** |
|  | (0.059) |  | (0.059) |  | (0.059) |  | (0.056) |  | (0.056) |  | (0.056) |  |
| Slovenia | -0.439 | *** | -0.441 | *** | -0.442 | *** | -0.151 | ** | -0.151 | ** | -0.156 | ** |
|  | (0.058) |  | (0.058) |  | (0.058) |  | (0.055) |  | (0.055) |  | (0.055) |  |
| Spain | -0.490 | *** | -0.491 | *** | -0.492 | *** | -0.504 | *** | -0.504 | *** | -0.505 | *** |
|  | (0.077) |  | (0.077) |  | (0.077) |  | (0.067) |  | (0.067) |  | (0.067) |  |
| Sweden | -0.532 | *** | -0.533 | *** | -0.534 | *** | -0.085 |  | -0.085 |  | -0.097 |  |
|  | (0.063) |  | (0.063) |  | (0.063) |  | (0.060) |  | (0.060) |  | (0.060) |  |
| Switzerland | -0.660 | *** | -0.660 | *** | -0.657 | *** | -0.243 | *** | -0.243 | *** | -0.242 | *** |
|  | (0.058) |  | (0.058) |  | (0.058) |  | (0.065) |  | (0.065) |  | (0.065) |  |
| Wave (ref: Wave 2) |  |  |  |  |  |  |  |  |  |  |  |  |
| Wave 4 | 0.063 | *** | 0.062 | *** | 0.063 | *** | 0.061 | *** | 0.061 | *** | 0.061 | *** |
|  | (0.015) |  | (0.015) |  | (0.015) |  | (0.014) |  | (0.014) |  | (0.014) |  |
| Wave 5 | 0.201 | *** | 0.201 | *** | 0.201 | *** | 0.222 | *** | 0.222 | *** | 0.221 | *** |
|  | (0.015) |  | (0.015) |  | (0.015) |  | (0.015) |  | (0.015) |  | (0.015) |  |
| Wave 6 | 0.337 | *** | 0.337 | *** | 0.337 | *** | 0.392 | *** | 0.392 | *** | 0.391 | *** |
|  | (0.016) |  | (0.016) |  | (0.016) |  | (0.016) |  | (0.016) |  | (0.016) |  |
| Wave 7 | 0.475 | *** | 0.476 | *** | 0.477 | *** | 0.585 | *** | 0.585 | *** | 0.588 | *** |
|  | (0.022) |  | (0.022) |  | (0.022) |  | (0.021) |  | (0.021) |  | (0.021) |  |
| Wave 8 | 0.512 | *** | 0.511 | *** | 0.511 | *** | 0.542 | *** | 0.542 | *** | 0.542 | *** |
|  | (0.018) |  | (0.018) |  | (0.018) |  | (0.018) |  | (0.018) |  | (0.018) |  |
| Wave 9 | 0.566 | *** | 0.566 | *** | 0.565 | *** | 0.566 | *** | 0.566 | *** | 0.567 | *** |
|  | (0.018) |  | (0.018) |  | (0.018) |  | (0.018) |  | (0.018) |  | (0.018) |  |
| Number of persons | 49,941 |  | 49,941 |  | 49,941 |  | 62,417 |  | 62,417 |  | 62,417 |  |
| Number of observations | 112,132 |  | 112,132 |  | 112,132 |  | 151,690 |  | 151,690 |  | 151,690 |  |
| R-squared | 0.180 |  | 0.181 |  | 0.181 |  | 0.214 |  | 0.214 |  | 0.215 |  |
| Inverse probability weights | 1.647 |  | 1.647 |  | 1.647 |  | 1.533 |  | 1.533 |  | 1.533 |  |
|  | (0.002) |  | (0.002) |  | (0.002) |  | (0.001) |  | (0.001) |  | (0.001) |  |

* p < 0.05; ** p < 0.01; *** p < 0.001

Notes: family w/ spouse = living with family and spouse/partner; family w/o spouse = living with family without spouse/partner

**Figure S1.** Predicted age-related profiles of chronic disease accumulation by migration background and living arrangements among men based on origin-stratified analysis. Origin countries are grouped based on the Human Development Index (HDI) into low (HDI < 0.7), medium (HDI ≥ 0.7), and high (HDI ≥ 0.8) development countries. Non-focal predictors are set to the average level across observations in the sample.

**Figure S2.** Predicted age-related profiles of chronic disease accumulation by migration background and living arrangements among women based on origin-stratified analysis. Origin countries are grouped based on the Human Development Index (HDI) into low (HDI < 0.7), medium (HDI ≥ 0.7), and high (HDI ≥ 0.8) development countries. Non-focal predictors are set to the average level across observations in the sample.

**Figure S3.** Predicted age-related profiles of chronic disease accumulation by migration background and living arrangements based on models with lagged living arrangements variable among (a) men and (b) women. Non-focal predictors are set to the average level across observations in the sample.

**Figure S4.** Predicted age-related profiles of chronic disease accumulation by migration background and living arrangements for (a) men and (b) women, in samples excluding immigrants who stayed less than 10 years in the receiving country. Non-focal predictors are set to the average level across observations in the sample.

**Figure S5.** Predicted number of chronic diseases by migration background and living arrangements in samples stratified by country of origin among (a) men and (b) women. Origin countries are grouped based on gross national income (GNI) per capita into low- (≤ US$ 4,035), medium- (> US$ 4,035 and ≤ US$ 12,475), and high-income (> US$ 12,475) economies. Non-focal predictors are set to the average level across observations in the sample.

**Figure S6.** Predicted number of chronic diseases by migration background and detailed living arrangements distinguishing living as a family with versus without spouse or partner from each model among (a) men and (b) women. Model 1 includes migration background, linear and quadratic terms of age, an interaction between migration background and age terms, and living arrangements. Model 2 adds an interaction term between migration background and living arrangements to Model 1. Model 3 adds an interaction term between migration background, age terms, and living arrangements to Model 2. All models are adjusted for country, wave, education, household income, employment status, and parental status. Non-focal predictors are set to the average level across observations in the sample.
